# Development of a novel high-throughput screen for the identification of new inhibitors of protein S-acylation

**DOI:** 10.1016/j.jbc.2022.102469

**Published:** 2022-09-08

**Authors:** Christine Salaun, Hiroya Takizawa, Alex Galindo, Kevin R. Munro, Jayde McLellan, Isamu Sugimoto, Tomotaka Okino, Nicholas C.O. Tomkinson, Luke H. Chamberlain

**Affiliations:** 1 Strathclyde Institute of Pharmacy and Biomedical Sciences, University of Strathclyde, Glasgow, United Kingdom; 2 Ono Pharmaceutical Co Ltd, Osaka, Japan; 3 Department of Pure and Applied Chemistry, University of Strathclyde, Glasgow, United Kingdom

**Keywords:** zDHHC enzymes, acyltransferase, protein acylation, protein palmitoylation, chemical inhibitor, high-throughput screening, click chemistry, PSD95, SNAP25, zDHHC2, APCI, atmospheric pressure chemical ionization, ARD, ankyrin repeat domain, 2BP, 2-bromopalmitic acid, BSA, bovine serum albumin, cDNA, complementary DNA, CRD, cysteine-rich domain, DDM, *n*-dodecyl-β-d-maltoside, DHHC, Asp-His-His-Cys, DMEM, Dulbecco's modified Eagle's medium, DMSO, dimethyl sulfoxide, EGFR, epidermal growth factor receptor, ESI, electrospray ionization, GST, glutathione-*S*-transferase, HA, hemagglutinin, HEK293T, human embryonic kidney 293T cell line, HRP, horseradish peroxidase, HTS, high-throughput screen, Lit, literature value, LRMS, low-resolution mass spectrometry, mp, melting point, NBD, nitrobenzoxadiazole, NEM, *N*-ethyl maleimide, NKA, Na/K ATPase, PSD95, postsynaptic density protein 95, SARS-CoV-2, severe acute respiratory syndrome coronavirus 2, STING, stimulator of interferon genes, TCEP, tris(2 carboxyethyl) phosphine hydrochloride, TR-FRET, time-resolved FRET, TTZ, tetrazole, zDHHC, zinc finger DHHC domain–containing protein

## Abstract

Protein S-acylation is a reversible post-translational modification that modulates the localization and function of many cellular proteins. S-acylation is mediated by a family of zinc finger DHHC (Asp-His-His-Cys) domain–containing (zDHHC) proteins encoded by 23 distinct *ZDHHC* genes in the human genome. These enzymes catalyze S-acylation in a two-step process involving “autoacylation” of the cysteine residue in the catalytic DHHC motif followed by transfer of the acyl chain to a substrate cysteine. S-acylation is essential for many fundamental physiological processes, and there is growing interest in zDHHC enzymes as novel drug targets for a range of disorders. However, there is currently a lack of chemical modulators of S-acylation either for use as tool compounds or for potential development for therapeutic purposes. Here, we developed and implemented a novel FRET-based high-throughput assay for the discovery of compounds that interfere with autoacylation of zDHHC2, an enzyme that is implicated in neuronal S-acylation pathways. Our screen of >350,000 compounds identified two related tetrazole-containing compounds (TTZ-1 and TTZ-2) that inhibited both zDHHC2 autoacylation and substrate S-acylation in cell-free systems. These compounds were also active in human embryonic kidney 293T cells, where they inhibited the S-acylation of two substrates (SNAP25 and PSD95 [postsynaptic density protein 95]) mediated by different zDHHC enzymes, with some apparent isoform selectivity. Furthermore, we confirmed activity of the hit compounds through resynthesis, which provided sufficient quantities of material for further investigations. The assays developed provide novel strategies to screen for zDHHC inhibitors, and the identified compounds add to the chemical toolbox for interrogating cellular activities of zDHHC enzymes in S-acylation.

S-acylation (or S-palmitoylation) is the post-translational attachment of long-chain fatty acids (predominantly palmitates) to cysteines *via* a labile thioester bond (1, 2, 3, 4). S-acylation is reversible, making it a dynamic post-translational modification that is able to regulate the stability, localization, activity, and function of a broad range of proteins. Around 10% of the human proteome might be modified by S-acylation (5), and this reaction is catalyzed by a family of 23 zinc finger Asp–His–His–Cys (DHHC) domain–containing (zDHHC) enzymes, defined by a 51-amino acid cysteine-rich domain (CRD) with a DHHC motif (6, 7, 8). zDHHC enzymes use a two-step ping–pong mechanism to mediate S-acylation (9, 10, 11). The first step is the autoacylation of the enzyme that involves the attack on the acyl-CoA by the catalytic cysteine within the DHHC sequence. The second step is the transfer of the acyl chain from the enzyme to a cysteine residue of the target substrate. The crystal structures of zDHHC20 and zDHHC15 have been reported recently (11, 12), showing that the four transmembrane domains of these enzymes arrange into a tepee-like structure, providing a hydrophobic cavity for the fatty acyl chain that becomes attached to the DHHC motif cysteine during the autoacylation step of the enzyme reaction. A preference of some zDHHC enzymes for specific fatty acid substrates was revealed by *in vitro* (9) and *in cellulo* studies (13) before being confirmed following the resolution of the crystal structure of zDHHC20 bound to a fatty acid chain (11, 12). The amino acid residues lining the hydrophobic cavity indeed act as a molecular ruler for the length of the fatty acid chain that can be accommodated. In addition to the catalytic DHHC domain, conserved PaCCT (palmitoyltransferase conserved C terminus) and TTxE (Thr-Thr-X-Glu) motifs in the C terminus of zDHHC20 were suggested to be important for enzyme function by forming key contacts with the catalytic site and other parts of the protein (8, 11, 14).

Analysis of the curated S-acylated proteome indicates that 41% of synaptic proteins may undergo S-acylation (15). Broad changes in S-acylation levels are induced by modulation of neuronal activity, highlighting a fundamental role of S-acylation in regulating synaptic functions (16). Alterations in the balance between neuronal excitation and inhibition are associated with several neuropsychiatric disorders, such as autism, epilepsy, and schizophrenia (17). Given the large number of neuronal substrates and the dynamic nature of S-acylation, it is not surprising that this modification has been linked with various neurological disorders (18, 19, 20, 21). Genome-wide association analyses have identified *ZDHHC2* as a candidate gene for schizophrenia (22, 23) as well as Parkinson’s disease (24). A targeted study identified rare mutations within the *ZDHHC2* coding sequence of Han Chinese patients suffering from schizophrenia (23). It was also suggested that the presence of zDHHC2 protects dopaminergic neurons from death during the course of Parkinson’s disease (25). There is strong evidence linking mutations in *ZDHHC9* (26, 27) with intellectual disability. A recent study revealed an elevated expression of zDHHC8 in the brain tissue of patients suffering from temporal lobe epilepsy (28), and there is also a possible (although still controversial) association between *ZDHHC8* polymorphism and schizophrenia (19, 21). S-acylation deficits have been suggested in other neurological disorders (such as Alzheimer’s disease or Huntington’s disease) although demonstrated contributions of zDHHC enzymes to these are still missing (19, 21). In addition to neurological disorders, there is also evidence linking zDHHC enzymes to diabetes and cancer (2).

A major obstacle to progress of the S-acylation field is a lack of selective chemical inhibitors or modulators of zDHHC enzymes (29, 30, 31, 32, 33). Three main lipid-based chemical compounds have long been used for their ability to inhibit S-acylation (Supplemental Fig. 1). The most widely used compound is 2-bromopalmitic acid (2BP), whereas cerulenin and tunicamycin have been used less frequently. However, none of these compounds is thought to be selective for a specific zDHHC enzyme(s), and many of the effects of these compounds may be entirely nonspecific. Tunicamycin is primarily known as an inhibitor of N-linked glycosylation, and cerulenin has been shown to inhibit various fatty acid synthases (34). Although 2BP has been shown to irreversibly block the activity of purified zDHHC enzymes (6, 35) and bind to zDHHC enzymes in cells (36, 37), it has also been found to be highly promiscuous (38), labeling hundreds of cellular proteins (36, 37) and therefore has potential pleiotropic effects on cell metabolism (29). An effort to develop a broad-spectrum inhibitor of zDHHC enzymes that would overcome the major drawbacks of 2BP led to the synthesis of *N*-cyanomethyl-*N*-myracrylamide, which utilizes an acrylamide warhead linked to the same fatty acid chain as 2BP (33, 39). This promising new compound has been shown to inhibit the activity of zDHHC20 *in vitro* (IC_50_ of 1.35 ± 0.26 μM) and of many zDHHC enzymes in cells with a low level of toxicity.

Several screens were developed in an attempt to find more specific inhibitors of zDHHC-mediated S-acylation (31, 33, 40, 41). Five (nonlipid based) compounds were identified from the DIVERSet collection (ChemBridge Corporation) for their ability to reduce peptide substrate S-acylation (40). However, further investigation showed that the potency and selectivity of four of these compounds were not recapitulated with purified enzymes and their cognate substrates. Only one of the compounds (compound V) directly (and reversibly) inhibited the acyltransferase activity of four different yeast and human zDHHC enzymes and may therefore be a general inhibitor of zDHHC enzymes (35). Inhibitors of the zDHHC enzyme Erf2 (the yeast ortholog of the mammalian zDHHC9 enzyme) were identified from a large scaffold-ranking library (41). The hit compounds against Erf2 were based on a *bis*-cyclic piperazine backbone. Interestingly, the identified compounds were recently shown to inhibit zDHHC9-mediated S-acylation of the spike protein of the severe acute respiratory syndrome coronavirus 2 (SARS-CoV-2), the virus responsible for the coronavirus disease 2019 pandemic, and to reduce virus infectivity (42). Protein S-acylation inhibition has indeed been identified as a therapeutic target against SARS-CoV-2 (43). Finally, Qiu *et al.* (44) recently reported the serendipitous discovery of artemisinin (an antimalarial sesquiterpene lactone) as a zDHHC6 covalent inhibitor that attenuates NRas (and other zDHHC6 substrates) S-acylation without altering global S-acylation, suggesting that the selective inhibition of S-acylation enzymes might be achievable.

To advance efforts to identify novel S-acylation inhibitors, we have developed a high-throughput FRET-based autoacylation assay to screen >350,000 compounds for activity against zDHHC2 autoacylation. Furthermore, selected hits were further assessed using novel *in vitro* and established cell-based click chemistry–based substrate S-acylation assays. Two related compounds were identified that displayed activity against zDHHC2 in all three of these different assay formats. The compounds also appear to display partial enzyme isoform selectivity in cell-based assays. This study thus expands the available repertoire of assay platforms and chemical inhibitors to advance S-acylation research.

## Results

### Development of a novel FRET-based high-throughput screen to identify chemical modulators of zDHHC2 autoacylation

**Figure 1:**
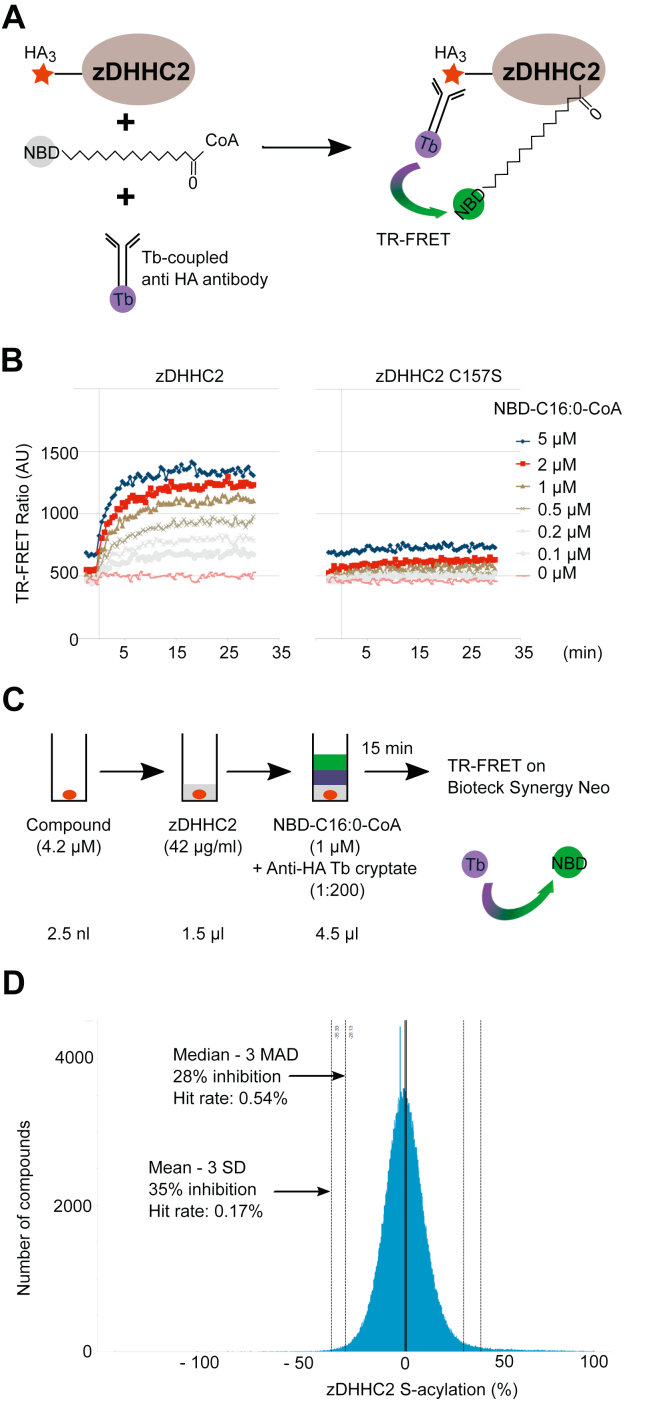
**High-throughput screen (HTS) for the discovery of chemical compounds modulating human zDHHC2 autoacylation activity: TR-FRET-based assay.***A*, experimental design of the HTS. HA-tagged zDHHC2 enzyme expressed at the surface of lipoparticles was incubated for 15 min at room temperature (RT) with NBD-tagged palmitoyl-CoA and an anti-HA antibody coupled to terbium (Tb) cryptate. Autoacylation of zDHHC2 on its catalytic cysteine brings closer the FRET donor Tb cryptate bound to the anti-HA antibody to the NBD fluorophore coupled to the palmitate and triggers FRET that can be followed over time (time-resolved FRET [TR-FRET]). *B*, TR-FRET was measured with increasing concentrations of NBD-palmitoyl-CoA (NBD-C16:0-CoA) and either WT zDHHC2 (*left panel*) or catalytically dead zDHHC2 C157S (*right panel*) over a period of 35 min. *C*, experimental conditions of the HTS screen. About 350,000 compounds were incubated individually in 1536-well plates at a concentration of 4.2 μM with zDHHC2 lipoparticles (42 μg/ml stock) for a few minutes before the addition of a mix containing the Tb cryptate–tagged anti-HA antibody (1:200 dilution) and 1 μM of NBD-palmitoyl-CoA (NBD-C16:0-CoA). TR-FRET was measured after a 15 min incubation at room temperature on a Biotek Synergy Neo apparatus. Results are given as a ratio between the FRET signal measured over a background baseline. *D*, histogram representation of ONO library screen showing the percentage of activity of zDHHC2 (as measured by TR-FRET) in the presence of each compound. The graph represents the number of compounds that are either activating (0 to 100) or inhibiting (0 to −100) zDHHC2 activity. HA, hemagglutinin; NBD, nitrobenzoxadiazole; TR-FRET, time-resolved FRET; zDHHC, zinc finger DHHC domain–containing protein.

S-acylation is a two-step process involving enzyme “autoacylation” (at the cysteine of the DHHC motif) followed by transfer of the acyl chain to a substrate protein. To identify potential inhibitors of S-acylation, we developed an assay measuring enzyme autoacylation and focused on zDHHC2 given its prominent role in synaptic function in the nervous system (Fig. 1*A*). The assay monitors time-resolved FRET (TR-FRET) between the energy donor terbium cryptate (attached to an antibody recognizing hemagglutinin [HA]-tagged zDHHC2) and the acceptor fluorophore nitrobenzoxadiazole (NBD) (attached to the lipid group). The excitation of the energy donor terbium cryptate (360 nm) followed by its emission at 480 nm results in energy transfer to the acceptor NBD and emission at 539 nm. Enzyme autoacylation by NBD-conjugated palmitoyl-CoA increases the proximity of the terbium cryptate and NBD, leading to an increase in FRET signal. The resulting interaction is quantified as the ratiometric measurement of NBD (539 nm) over terbium (480 nm) emission and constitutes the TR-FRET signal. Human zDHHC2 enzyme for use in this assay was produced with the Expi293 MembranePro expression system (Invitrogen), which enables membrane proteins to be displayed in a native context on highly enriched lipoparticles that are released in the supernatant of the transfected mammalian cells.

An example of the TR-FRET measured using this assay system is shown in Fig. 1*B*. Autoacylation of zDHHC2, detected by TR-FRET signal, was shown to increase with increasing concentration of NBD-palmitoyl-CoA. Importantly, the TR-FRET signal was substantially reduced when a catalytically dead form of zDHHC2 with a substitution of the cysteine in the DHHC motif (C157S) was used, demonstrating that the FRET signal faithfully reports the autoacylation of the enzyme.

**Figure 2:**
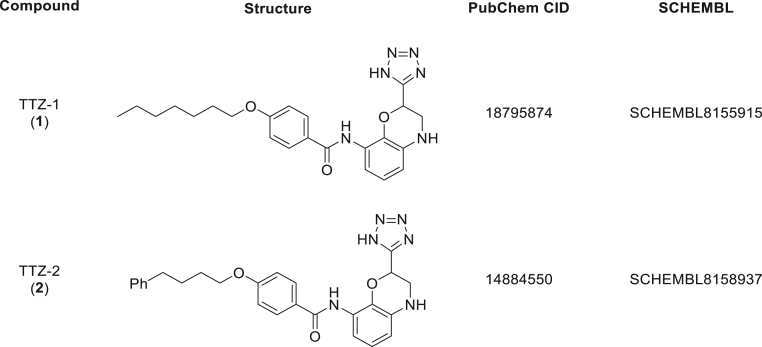
**Chemical structure of TTZ-1 and TTZ-2.** TTZ, tetrazole.

Following optimization, the assay was miniaturized to a 1536-well plate format. A pilot screen was then performed at two concentrations (12.5 and 4.2 μM) using ONO pilot library containing a total of 15,792 compounds. RZ′ score was calculated to be between 0.72 and 0.80 for each plate showing that the assay design was robust. Fig. 1*C* outlines the final conditions chosen for the screen, which were then used to screen a high-throughput screen (HTS) library of >350,000 compounds at a concentration of 4.2 μM (Fig. 1*D*). About 1600 compounds that presented an inhibition equal to the median – 3 SD or less were then examined in duplicate and at two concentrations. Following this, we selected the 162 most potent compounds and again assessed their inhibitory activity by TR-FRET using freshly dissolved powder and at two different concentrations (12.5 and 4.2 μM). Their ability to affect substrate S-acylation was also assessed using the *in vitro* ELISA described later at two different concentrations (3 and 10 μM) and in duplicate experiments. Although several compounds were confirmed to have an activity *in vitro*, the results presented focus on two of these compounds (tetrazole [TTZ] -1 and -2) because they were subsequently found to have the highest inhibitory activity on S-acylation in cell-based assays (see later). The chemical structure of these two compounds is shown in Fig. 2. Table 1 presents data obtained from the HTS and confirmation screens for those compounds, which shows that zDHHC2 autoacylation was inhibited by 61% by TTZ-1 and 69% by TTZ-2 at a concentration of 12.5 μM.

**Table 1:** Summary of data obtained at both research sites for compounds TTZ-1 and TTZ-2

	*In vitro*			
	zDHHC2 autoacylation		zDHHC2-mediated SNAP25	
	inhibition (%)		S-acylation inhibition (%)	
Location	Ono		UoS	
Technique	TR-FRET		Copper-free click chemistry	
			Biotinylated probe	
			ELISA	
Compound concentration (μM)	12.5	4.2	10	3
TTZ-1	61	32	77	30
TTZ-2	69	45	81	64


	*In cellulo*				
	SNAP25 S-acylation inhibition (%)				
Location	UoS				
Technique	Copper-catalyzed click chemistry				
	Fluorescent probe				
	Western blot				
Compound concentration (μM)	25	25	25	25	25
	zDHHC2	zDHHC3	zDHHC7	zDHHC15	zDHHC17
TTZ-1	113 ± 5	9 ± 4	14 ± 7	112 ± 6	36 ± 7
TTZ-2	84 ± 6	19 ± 3	13 ± 7	93 ± 6	39 ± 7


	*In vitro*			*In cellulo*		
	zDHHC2-mediated substrate (SNAP25) S-acylation					
Location	UoS			UoS		
Technique	Copper-free click chemistry			Copper-catalyzed click chemistry		
	Biotinylated probe			Fluorescent probe		
	ELISA			Western blot		
	IC_50_ (95 CI) (μM)	Hill slope	*R*^2^	IC_50_ (95 CI) (μM)	Hill slope	*R*^2^
TTZ-1	4.7 (3.6–6.1)	4.7	0.97	14.4 (10.2–20.3)	7.6	0.80
TTZ-2	1.3 (0.8–2.2)	1.3	0.88	18.8 (15.1–23.4)	4.6	0.79

The table gives the location of the research (either ONO or University of Strathclyde UoS), the technique used, and the numerical data generated, both for *in vitro* and in HEK293T cells (*in cellulo*).

### Development of a novel ELISA to detect *in vitro* substrate S-acylation by purified human zDHHC2

**Figure 3:**
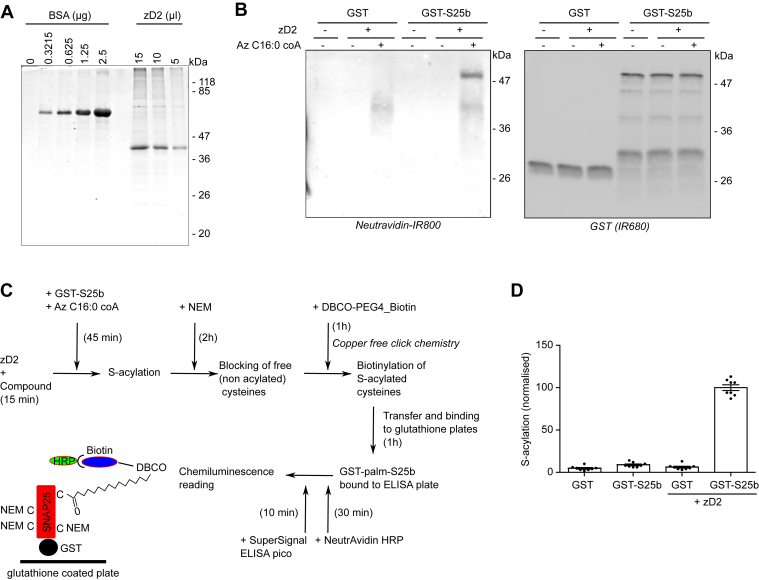
**Purification of active human zDHHC2 enzyme from insect cells and development of an ELISA for the detection of substrate S-acylation.***A*, purification of human zDHHC2. His-tagged human zDHHC2 (zD2) was expressed with a baculovirus system in Sf9 TriEX insect cells and purified using Ni^2+^–nitrilotriacetic acid resin. Aliquots were resolved on a SDS-PAGE gel together with increasing amounts of bovine serum albumin (BSA) standards. Proteins were revealed with a staining agent (Instant Blue). The main band migrating between 36 and 47 kDa is at the estimated size for tagged zDHHC2. Positions of the molecular mass markers (in kilodalton) are indicated on the *right side* of the gel. *B*, *in vitro* S-acylation with purified zDHHC2 and detection by copper-free click chemistry. The zDHHC2 substrate GST-SNAP25b (GST-S25b) or a control GST protein (both at 1 μM final) were incubated either with (+) or without (−) zDHHC2 (zD2) (0.075 μM final) and either with (+) or without (−) 1 μM azide-C16:0-CoA (Az-C16:0-CoA) in acylation buffer for 45 min at 25 °C. Free (nonacylated cysteines) were then blocked for 2 h with 50 mM NEM. Copper-free click chemistry was then started by the addition of 5 μM DBCO-PEG_4_-Biotin in PBS. Samples were incubated for 1 h at room temperature and diluted five times by the addition of PBS before being resolved on a SDS-PAGE gel and transferred to a nitrocellulose membrane. Biotinylated proteins were revealed by incubating the membrane with NeutrAvidin-IR800, whereas the GST-tagged proteins were detected with an anti-GST antibody (IR680). Positions of the molecular mass markers (in kilodalton) are indicated on the *right side* of the membranes. *C*, schematics of the ELISA protocol. *D*, samples were treated as in *B* and analyzed with the S-acylation ELISA as schematized in *C*. The graph represents the mean ± SEM of the normalized ELISA values. Each *circle* represents one value; values were gathered from four independent *in vitro* S-acylation experiments performed in duplicate. GST, glutathione-*S*-transferase; NEM, *N*-ethyl maleimide; zDHHC, zinc finger DHHC domain–containing protein.

We next assessed the ability of TTZ-1 and TTZ-2 to inhibit zDHHC2-mediated substrate S-acylation *in vitro*. For this, we designed an ELISA in a 96-well plate format. His_6_-tagged human zDHHC2 enzyme was purified from insect cells on Ni^2+^–nitrilotriacetic acid affinity resin according to methods developed previously (9) (Fig. 3*A*). Glutathione-*S*-transferase (GST)-tagged SNAP25, a reported substrate of zDHHC2 (45), was then incubated with the purified zDHHC2 enzyme and azide-C16:0-CoA for 30 to 45 min (GST was used as a control). After treatment with 50 mM *N*-ethyl maleimide (NEM) for 2 h to block free cysteines, the azide-tagged palmitate that had been incorporated into GST-SNAP25 and zDHHC2 was reacted with DBCO-PEG4-Biotin in a copper-free cycloaddition reaction. Samples were then analyzed by immunoblotting with NeutrAvidin-800 to detect biotin (and hence S-acylation) and an anti-GST antibody (IR680) to detect GST and GST-SNAP25. Fig. 3*B* shows that this assay detects the S-acylation of GST-SNAP25 (band above 47 kDa) but not GST. The NeutrAvidin-800 signal also revealed the autoacylated enzyme (band above 36 kDa).

This assay was further developed into an ELISA format to more precisely quantify SNAP25 S-acylation (Fig. 3*C*). *In vitro* S-acylation and click chemistry labeling were carried out in uncoated 96-well plates. The processed samples were then diluted with PBS and bound for 1 h to glutathione plates before being incubated with NeutrAvidin-peroxidase (horseradish peroxidase [HRP]). Chemiluminescence signal was then measured following a 10 min incubation with a peroxidase substrate. Compounds tested in this ELISA were preincubated with the purified zDHHC2 enzyme for 15 min at room temperature prior to the addition of substrates. Fig. 3*D* shows the normalized chemiluminescent signal detected with the ELISA. Background levels of signal were detected with GST or GST-SNAP25 alone (with no added zDHHC2) (4.8 ± 0.9 and 8.9 ± 1.0, respectively) or with GST + zDHHC2 (6.1 ± 1.1), whereas there was a strong signal for samples incubated with GST-SNAP25 and the purified enzyme (100 ± 3.4). The ELISA was therefore suitable for testing the activity of the identified compounds on the S-acylation of SNAP-25 by purified zDHHC2.

### TTZ-1 and TTZ-2 inhibit zDHHC2-mediated SNAP25 S-acylation *in vitro* with IC_50_ values below 5 μM

**Figure 4:**
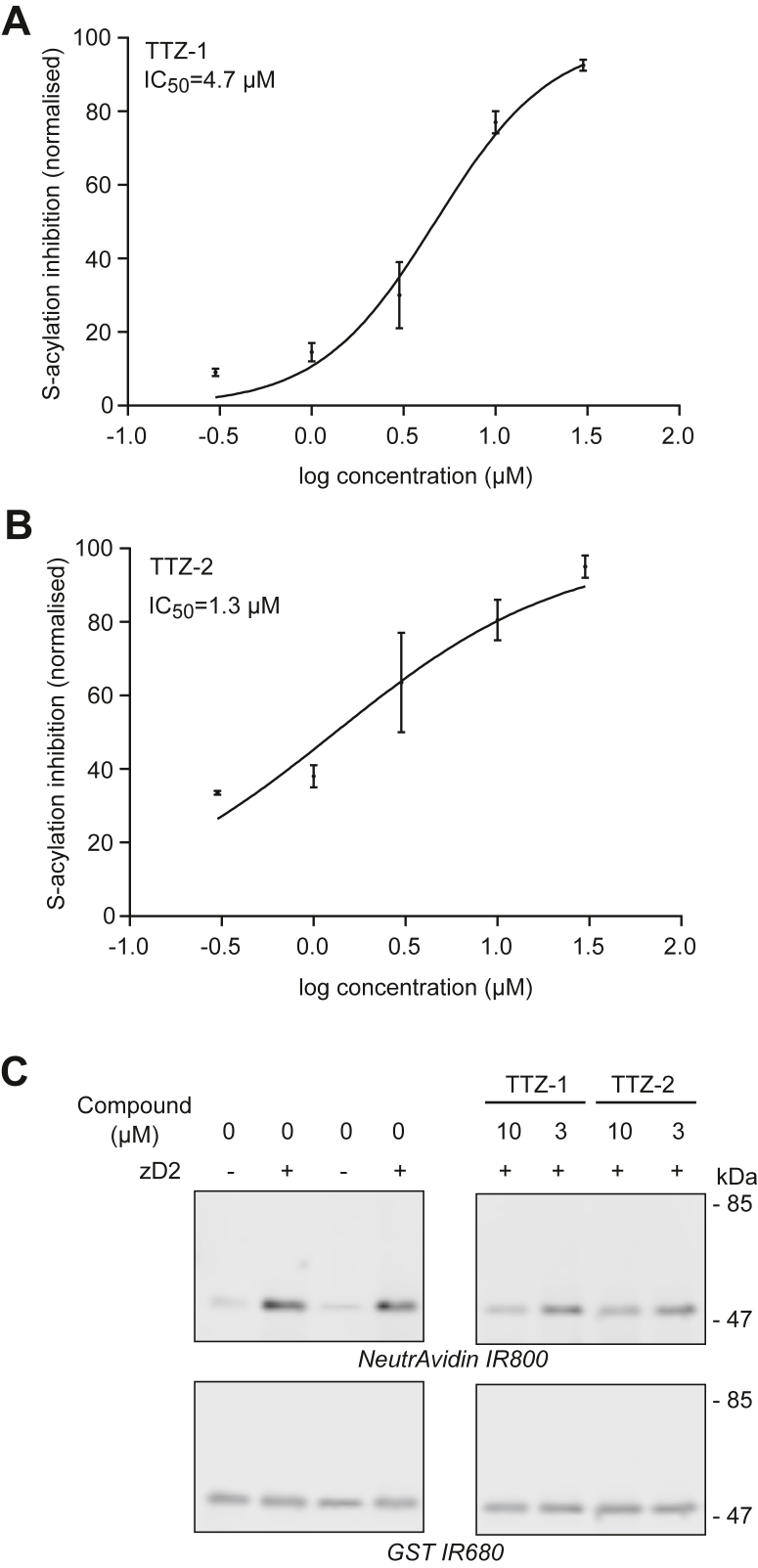
**Activity of TTZ-1 and TTZ-2 against SNAP25 S-acylation by purified human zDHHC2 *in vitro.*** Increasing concentrations of TTZ-1 (*A*) and TTZ-2 (*B*) compounds or DMSO (as a control) were preincubated with purified zDHHC2 (zD2) for 15 min at room temperature. Azide-C16:0-CoA and GST-SNAP25b were then added, and S-acylation was measured as described for Fig. 3*C*. The mean ELISA value of a control sample (GST-SNAP25b without zDHHC2; basal background values of the ELISA) was subtracted from all the values that were then normalized to the mean value of the control sample GST-SNAP25b + zDHHC2 treated with DMSO. Data shown on the graphs are mean ± SEM of the percentage of inhibition of zDHHC2-mediated GST-SNAP25b S-acylation for two independent experiments. Data were analyzed and fit to a nonlinear curve using the log(inhibitor) *versus* normalized response (variable slope) equation (GraphPad). *C*, selected samples from the S-acylation reactions from *A* and *B* were resolved on an SDS-PAGE gel, transferred to a nitrocellulose membrane, and probed with NeutrAvidin 800 and an anti-GST antibody (IR 680). Molecular mass markers are indicated on the *right side* of the blots. The immunoblots shown are from the same membrane but have lanes removed that contain samples that are not relevant to this study. DMSO, dimethyl sulfoxide; GST, glutathione-*S*-transferase; TTZ, tetrazole; zDHHC, zinc finger DHHC domain–containing protein.

The ability of TTZ-1 and TTZ-2 to inhibit SNAP25 S-acylation by purified zDHHC2 *in vitro* was then determined by calculating IC_50_ values (with concentrations ranging from 0.3 to 30 μM). Fig. 4 shows that both compounds inhibited S-acylation of SNAP25 by zDHHC2 with an IC_50_ below 5 μM (Table 1). Selected samples were also analyzed by SDS-PAGE and Western blotting, which confirmed the inhibitory activity of both compounds (at 3 or 10 μM) on SNAP25 S-acylation (Fig. 4*C*). Importantly, this confirms that the decreased signal detected with these compounds in the ELISA reliably reports on reduced S-acylation rather than any nonspecific effects.

### TTZ-1 and TTZ-2 inhibit zDHHC2-mediated SNAP25 S-acylation in cells with IC_50_ values below 20 μM

**Figure 5:**
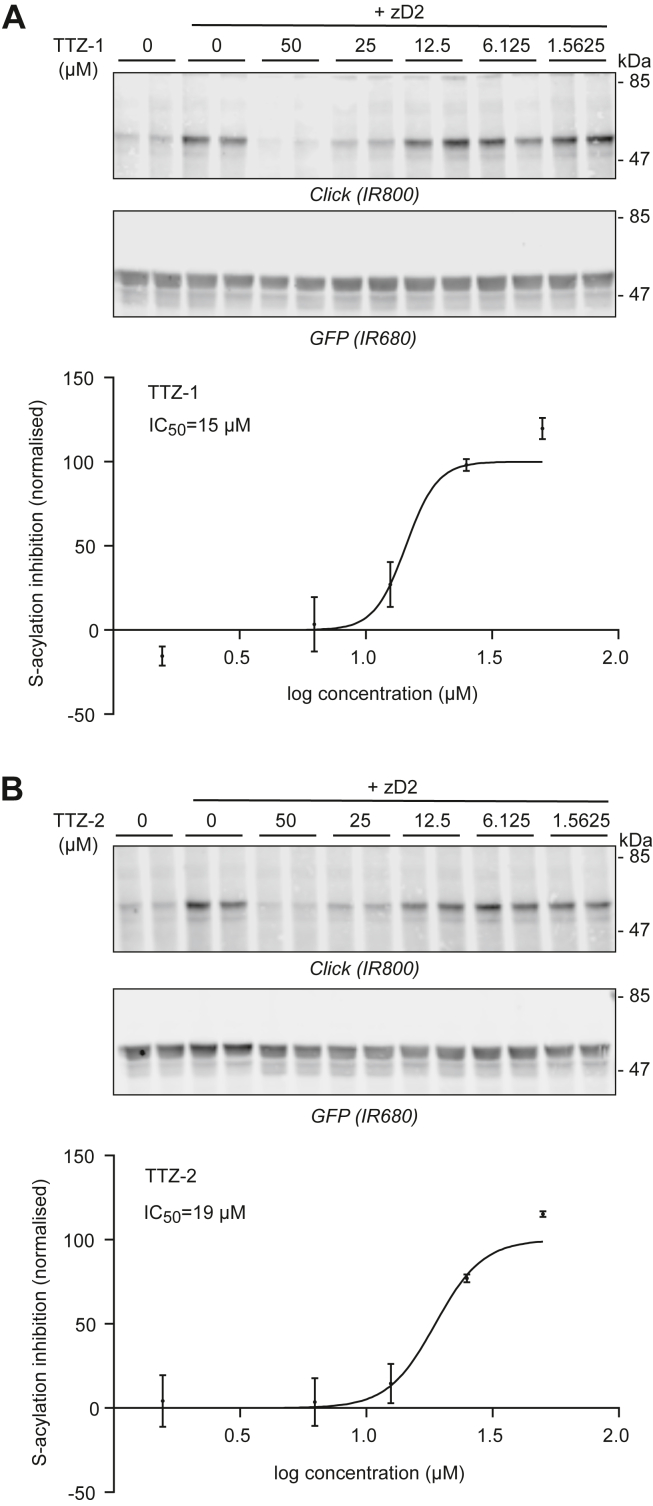
**Activity of TTZ-1 and TTZ-2 against SNAP25 S-acylation by human zDHHC2 expressed in mammalian cells.** HEK293T cells were transfected with a plasmid encoding GFP-SNAP25b together with a plasmid encoding human zDHHC2 (+zD2) or a control plasmid pEF-BOS-HA. Cells were preincubated for 4 h with increasing concentrations of TTZ-1 (*A*) or TTZ-2 (*B*) or DMSO (as a control) before metabolic labeling with 100 μM C16:0-azide for another 4 h. Cells were lysed, and proteins having incorporated C16:0-azide were labeled by click chemistry with an alkyne-IR800 dye. Protein samples were then resolved by SDS-PAGE and transferred to nitrocellulose membranes that were probed with an anti-GFP antibody (IR680). Representative images are shown for cells treated with TTZ-1 (*A*) or TTZ-2 (*B*). *Top panels*, click chemistry signal (*top panel*, Click [IR800]) and GFP immunoblot (*middle panel*, GFP [IR680]). Positions of the molecular mass markers (in kilodalton) are indicated on the *right side* of the membranes. *Bottom panels A* and *B*, data from three independent experiments performed in duplicate were quantified, and the inhibition of GFP-SNAP25b S-acylation was determined as stated in the Experimental procedures section. Graphs show mean ± SEM of inhibition of normalized S-acylation (click signal/GFP signal) in the presence of each compound. Data were analyzed and fit to a nonlinear curve using the log(inhibitor) *versus* normalized response (variable slope) equation. DMSO, dimethyl sulfoxide; HA, hemagglutinin; HEK293T, human embryonic kidney 293T cell line; TTZ, tetrazole; zDHHC, zinc finger DHHC domain–containing protein.

The inhibitory activity of TTZ-1 and TTZ-2 on cells expressing SNAP25 and zDHHC2 was also examined. Human embryonic kidney 293T (HEK293T) cells were transfected with plasmids encoding GFP-tagged SNAP25b and HA-tagged zDHHC2. Cells were then preincubated for 4 h with either dimethyl sulfoxide (DMSO) as a vehicle control or increasing concentrations of TTZ-1/TTZ-2 (diluted in serum-free medium) before being metabolically labeled for a further 4 h with C16:0-azide. Samples were then lysed and processed by copper-catalyzed click chemistry to reveal the extent of S-acylation. Fig. 5, *A* and *B* shows that both compounds inhibited SNAP25 S-acylation catalyzed by zDHHC2, with IC_50_ values of approximately 15 μM for TTZ-1 and 19 μM for TTZ-2.

### Inhibition profile of TTZ-1 and TTZ-2 toward different zDHHC enzyme isoforms in cell-based S-acylation assays

**Figure 6:**
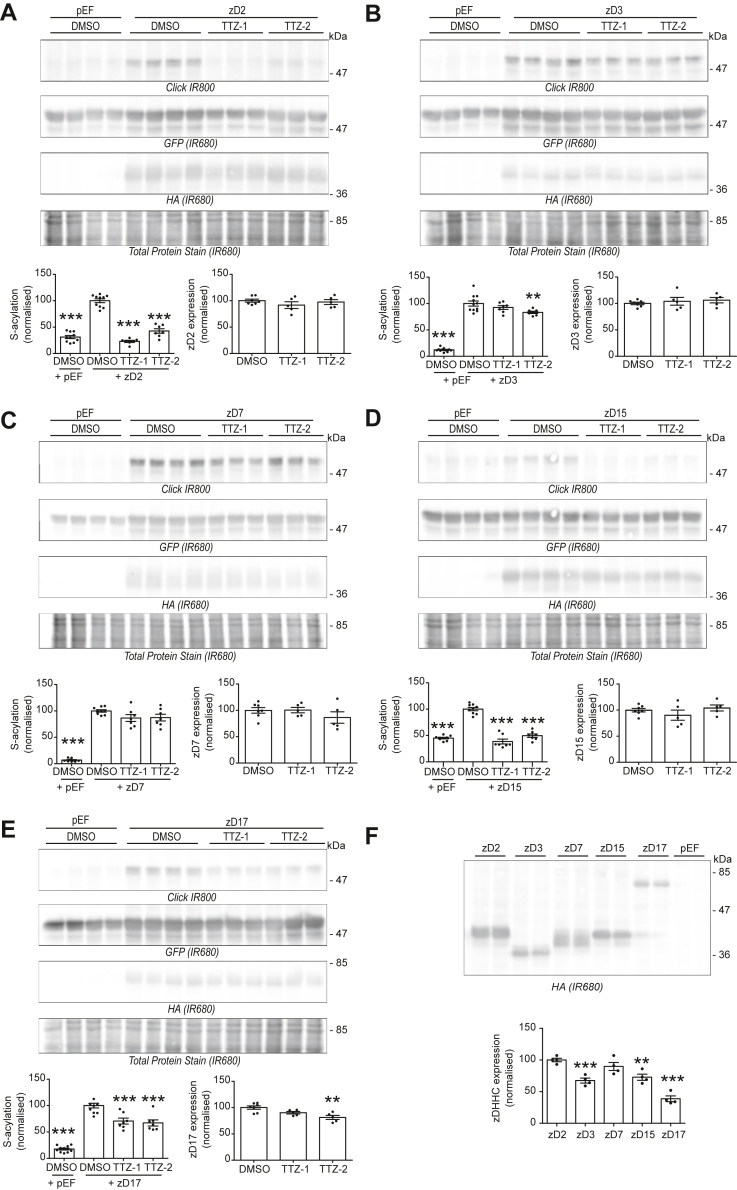
**Activity of TTZ-1 and TTZ-2 against SNAP25 S-acylation by human zDHHC2, zDHHC3, zDHHC7, zDHHC15, and zDHHC17 expressed in mammalian cells.***A*–*E*, HEK293T cells were transfected with a plasmid encoding GFP-SNAP25b together with either a control plasmid pEF-BOS-HA (pEF) or a plasmid encoding either HA-tagged human zDHHC2 (zD2) (*A*), zDHHC3 (zD3) (*B*), zDHHC7 (zD7) (*C*), zDHHC15 (zD15) (*D*), or zDHHC17 (zD17) (*E*) for 24 h. Cells were then preincubated for 4 h with TTZ-1 or TTZ-2 (25 μM final) or DMSO control before metabolic labeling with 100 μM C16:0-azide for 4 h. Cells were lysed, and proteins having incorporated C16:0-azide were labeled by click chemistry with an alkyne-IR800 dye. Protein samples were then resolved by SDS-PAGE and transferred to nitrocellulose membranes that were first stained with an infrared-680 total protein stain. Membranes were then destained and probed with an anti-HA antibody (IR680), followed by an anti-GFP antibody (IR680). Representative images are shown, and panels are presented in the following order (from *top* to *bottom*): click chemistry signal (Click [IR800]), GFP and HA immunoblots (IR680), and Total Protein Stain (IR680). Positions of the molecular mass markers (in kilodalton) are indicated on the *right side* of the membranes. Three independent experiments were performed either in duplicate or in triplicate, and the quantification data were gathered on the graphs below each set of membranes. The graph on the *left* shows mean ± SEM of normalized S-acylation of GFP-SNAP25b (click signal/GFP signal) for three independent experiments. *Filled circles* represent individual samples. Statistical analysis (ANOVA) was performed to reveal whether there was a significant difference between the zDHHC enzyme-mediated S-acylation of GFP SNAP25-treated with DMSO *versus* similar samples treated with TTZ-1 or TTZ-2 or *versus* cells that do not overexpress any zDHHC (+pEF) (∗∗∗*p* < 0.001; ∗∗*p* < 0.01). The graph on the *right* shows mean ± SEM of normalized zDHHC expression (HA signal/Total Protein Stain signal) from two independent experiments. *Filled circles* represent individual samples. Statistical analysis (ANOVA) was performed to test whether there was a significant difference between the zDHHC expression of cells treated with DMSO as a control *versus* similar samples treated with TTZ-1 or TTZ-2 (∗∗*p* < 0.01). *F*, samples from *A*, *B*, *C*, *D*, and *E* (expressing GFP-SNAP25 and HA-tagged zDHHC enzymes and treated with DMSO) were loaded on an SDS-PAGE gel and transferred to a nitrocellulose membrane. The membrane was probed for the expression of the HA-tagged enzymes with an anti-HA antibody (IR680). Positions of the molecular mass markers (in kilodalton) are indicated on the *right side* of the membranes. The graph represents the mean ± SEM of normalized zDHHC expression (HA signal) from two independent experiments. *Filled circles* represent individual samples. DMSO, dimethyl sulfoxide; HA, hemagglutinin; HEK293T, human embryonic kidney 293T cell line; TTZ, tetrazole; zDHHC, zinc finger DHHC domain–containing protein.

We next investigated whether the identified compounds were effective at inhibiting zDHHC enzyme isoforms other than zDHHC2 (Fig. 6 and Table 1). In addition to zDHHC2, SNAP25 has been shown to be a substrate for S-acylation by zDHHC3, zDHHC7, zDHHC15, and zDHHC17 when coexpressed in HEK293T cells (6, 45). We therefore treated cells coexpressing GFP-SNAP25 and each of these zDHHC enzymes with 25 μM of TTZ-1 and TTZ-2 and studied the subsequent effect on SNAP25 S-acylation. Fig. 6*A* shows complete inhibition of zDHHC2-mediated SNAP25 S-acylation by TTZ-1 (113 ± 5%) and a very strong reduction (84 ± 6%) by TTZ-2. Importantly, the level of enzyme expression was unaffected by the compounds. Interestingly, the inhibitory effect of the compounds on zDHHC15-mediated S-acylation of SNAP25 was similar to that seen with zDHHC2 (Fig. 6*D*). In contrast, S-acylation of SNAP25 by zDHHC3 and zDHHC7 was less inhibited: TTZ-1 had no significant effect on either enzyme in this assay, and TTZ-2 caused a 19 ± 3% reduction of SNAP25 S-acylation by zDHHC3 and had no significant effect on S-acylation by zDHHC7 (Fig. 6, *B* and *C*). Finally, both compounds showed an intermediate effect on zDHHC17-mediated S-acylation of SNAP25, with a ∼35 to 40% reduction (Fig. 6*E*). In addition, there was a slight but significant reduction in the level of zDHHC17 expressed in cells treated with TTZ-2. As any major differences in the expression levels of the different enzymes could in theory contribute to the apparent differential effects of TTZ-1 and TTZ-2, we directly compared the expression of zDHHC2, zDHHC3, zDHHC7, zDHHC15, and zDHHC17 when transfected in HEK293T cells together with GFP-SNAP25. As shown in Fig. 6*F*, none of the zDHHC enzymes was expressed to a higher level than HA-zDHHC2, suggesting that the observed differential effects of TTZ-1 and TTZ-2 are not because of increased expression levels of a particular enzyme.

### Synthesis of TTZ-1 and TTZ-2 confirms the identity of the hit compounds and allows confirmation of biological results

**Figure 7:**
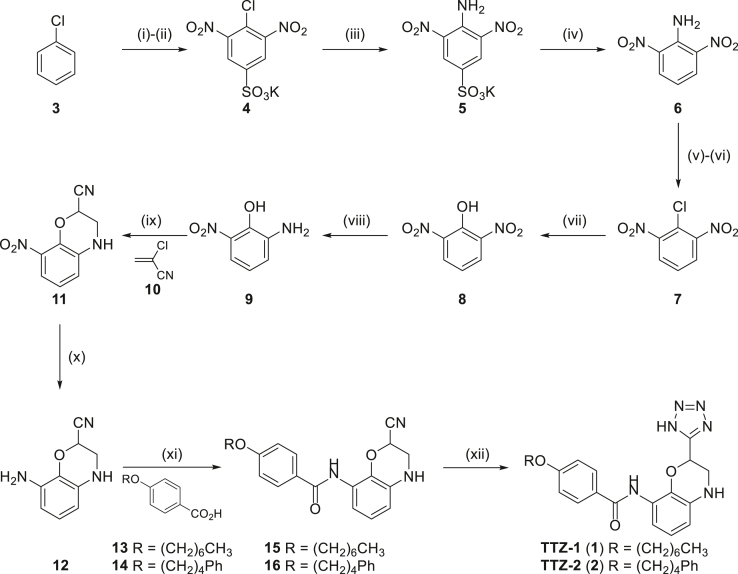
**Synthesis of TTZ-1** (1) **and TTZ-2** (2)**.***Reagents and conditions*: (i) H_2_SO_4(conc)_, 120 °C, 4 h. (ii) KNO_3_, 115 °C, 20 h. (iii) NH_3(aq)_, Δ, 1 h. (iv) H_2_SO_4(conc)_:H_2_O (1:1), Δ, 16 °C (36%, four steps). (v) NaNO_2_, H_2_SO_4_, 40 °C, 1 h. (vi) CuCl, HCl_(conc)_, 80 °C, 20 min (57%, two steps). (vii) KOH, H_2_O, Δ, 1 h (quant). (viii) 10% Pd/C, H_2_, MeOH, r.t., 1 h (88%). (ix) 10 (8.0 equiv.), Cs_2_CO_3_, PhMe, Δ, 4 h (15%). (x) 10% Pd/C, H_2_, MeOH, r.t., 1 h (70%). (xi) **13** (1 equiv.) or **14** (1 equiv.), HATU, DIPEA, DMF, r.t., 16 h (**15** 58%; **16** 45%). (xii) NaN_3_, NEt_3_·HCl, DMF, 140 °C, 2 h (**1** quant; **2** quant). TTZ, tetrazole.

To confirm the identity of the hit compounds and to allow further investigations to take place, we prepared samples of TTZ-1 and TTZ-2 through the multistep synthetic sequence outlined in Fig. 7. One-pot sulfonation/*bis*-nitration of chlorobenzene **3** led to the sulfonate salt **4**. S_N_Ar reaction with aqueous ammonia followed by protodesulfonation gave 2,6-dinotroaniline **6** in 36% overall yield for the initial sequence. Sandmeyer reaction of **6** under standard conditions gave 2,6-dinitrochlorobenzene **7** (57%), which was subjected to an efficient S_N_Ar reaction with hydroxide followed by partial reduction and then reacted with 2-chloroacrylonitrile **10** to give benzoxazine **11** in a poor, but acceptable, yield of 15% after purification by silica gel chromatography. Reduction of the second nitro functionality led to the central scaffold of the target ligand **12** (70%) from which diversity could be introduced. Amide bond coupling followed by acid-catalyzed TTZ formation gave TTZ-1 and TTZ-2. Compounds TTZ-1 and TTZ-2 were prepared in 12 synthetic steps and an overall yield of 1.9% and 1.5%, respectively.

**Figure 8:**
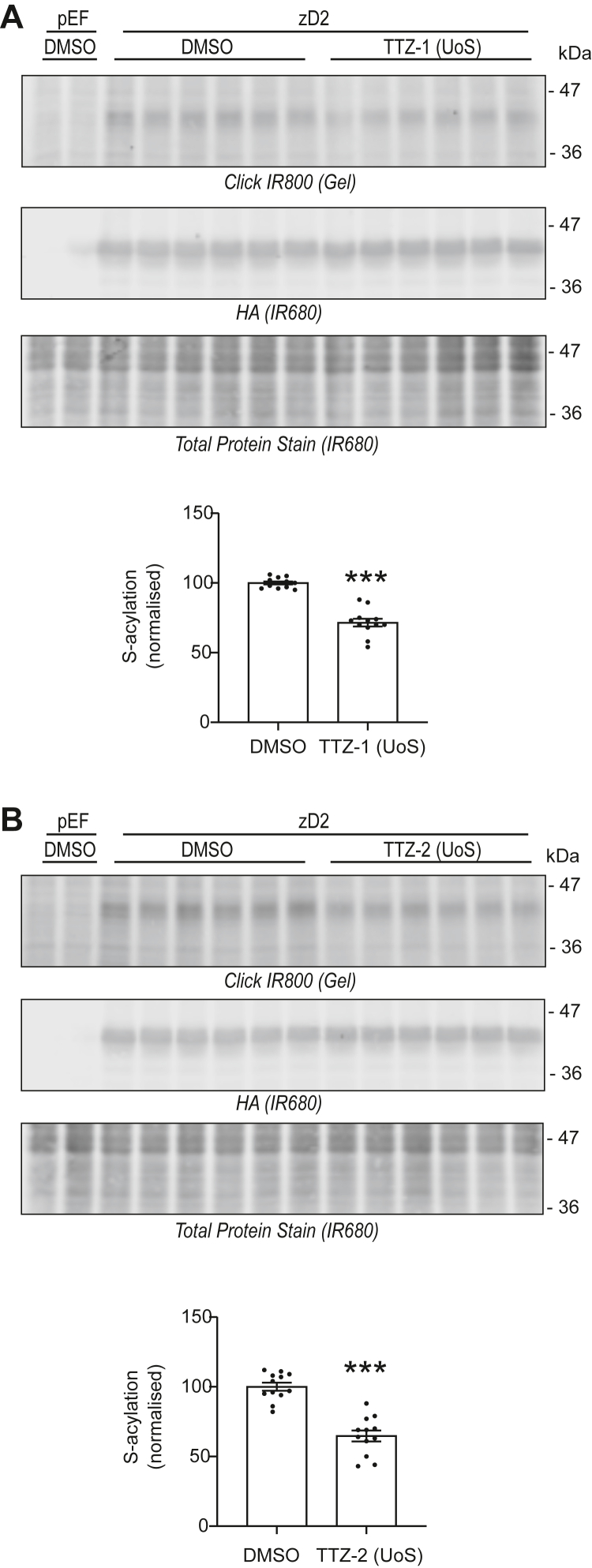
**Activity of TTZ-1 (UoS) and TTZ-2 (UoS) against human zDHHC2 autoacylation expressed in mammalian cells.** HEK293T cells were transfected with either the control plasmid pEF-BOS-HA (pEF) or a plasmid encoding HA-tagged human zDHHC2 (zD2) for 24 h. Cells were then preincubated for 4 h with UoS compounds TTZ-1 (UoS) (*A*) or TTZ-2 (UoS) (*B*) (25 μM final) or DMSO control before metabolic labeling with 100 μM C16:0-azide for 4 h. Cells were lysed, and proteins having incorporated C16:0-azide were labeled by click chemistry with an alkyne-IR800 dye. Protein samples were then resolved by SDS-PAGE, and the gel was scanned for the detection of the click chemistry signal in the infrared-800 channel. Gels were then transferred to nitrocellulose membranes that were first stained with an infrared-680 total protein stain. Membranes were then destained and probed with an anti-HA antibody (IR680). Representative images are shown, and panels are presented in the following order (from *top* to *bottom*): click chemistry signal within the gel (Click [IR800] [Gel]), HA immunoblots (IR680), and Total Protein Stain (IR680). Positions of the molecular mass markers (in kilodalton) are indicated on the *right side* of the membranes. Two independent experiments were performed in sextuplicate, and the quantification data were gathered on the graphs below each set of membranes. They show mean ± SEM of normalized S-acylation of HA-zDHHC2 (click signal/HA signal). *Filled circles* represent individual samples. Statistical analysis (Student’s *t* test) reveals that there is a significant difference between the S-acylation of zDHHC2 in cells treated with DMSO *versus* similar samples treated with TTZ-1 or TTZ-2 (∗∗∗*p* < 0.001). DMSO, dimethyl sulfoxide; HA, hemagglutinin; HEK293T, human embryonic kidney 293T cell line; TTZ, tetrazole; zDHHC, zinc finger DHHC domain–containing protein.

The newly synthesized TTZ-1 and TTZ-2 compounds were then tested against GFP-SNAP25 S-acylation in cell-based assays with results comparable to the original hit compounds (Supplemental Fig. 2). We also tested the effects of these compounds on zDHHC2 S-acylation in HEK293T cells to confirm that the compounds affected autoacylation in cells as seen in the original HTS using purified components (Fig. 1). As shown in Fig. 8, both compounds significantly reduced the S-acylation of HA-zDHHC2 in HEK293T cells.

### Inhibition of postsynaptic density protein 95 S-acylation by TTZ-1 and TTZ-2

**Figure 9:**
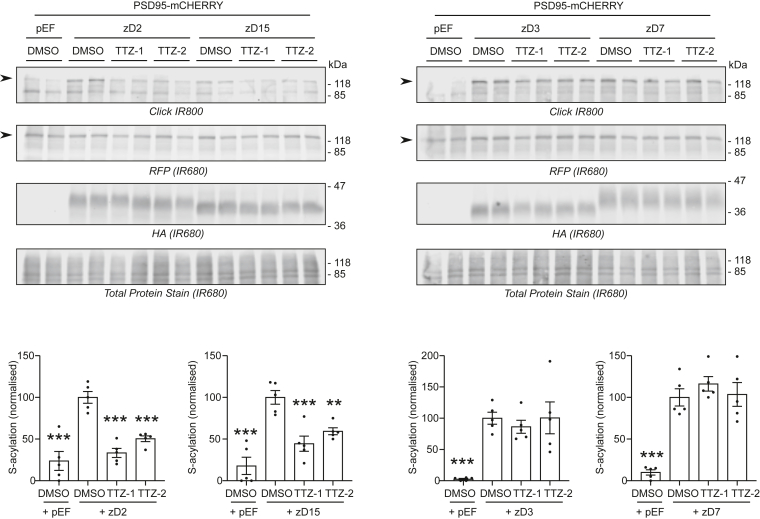
**Activity of TTZ-1 and TTZ-2 against PSD95 S-acylation mediated by human zDHHC2, zDHHC3, zDHHC7, and zDHHC15 expressed in mammalian cells.** HEK293T cells were transfected with a plasmid encoding PSD95-mCHERRY together with either a control plasmid pEF-BOS-HA (pEF) or a plasmid encoding either HA-tagged human zDHHC2 (zD2), zDHHC3 (zD3), zDHHC7 (zD7), or zDHHC15 (zD15) for 24 h. Cells were then preincubated for 4 h with TTZ-1 or TTZ-2 (25 μM final) or DMSO control before metabolic labeling with 100 μM C16:0-azide for 4 h. Cells were lysed, and proteins having incorporated C16:0-azide were labeled by click chemistry with an alkyne-IR800 dye. Protein samples were then resolved by SDS-PAGE and transferred to nitrocellulose membranes that were first stained with an infrared-680 total protein stain. Membranes were then destained and cut above the 47 kDa marker. The *topparts* of the membranes were probed with an anti-RFP antibody (recognizing the mCHERRY tag) (IR680), whereas the *bottom parts* were probed with an anti-HA antibody (IR680). Representative images are shown, and panels are presented in the following order (from *top* to *bottom*): click chemistry signal (Click [IR800]), RFP, and HA immunoblots (IR680), and Total Protein Stain (IR680). Positions of the molecular mass markers (in kilodalton) are indicated on the *right side* of the membranes. Two independent experiments were performed either in duplicate or in triplicate. The data were quantified, and the graphs below each set of membranes show mean ± SEM of normalized S-acylation of PSD95-mCHERRY (click signal/RFP signal). *Filled circles* represent individual samples. Statistical analysis (ANOVA) was performed to reveal whether there was a significant difference between the zDHHC enzyme-mediated S-acylation of PSD95-mCHERRY treated with DMSO *versus* similar samples treated with TTZ-1 or TTZ-2 or *versus* cells that do not overexpress any zDHHC (+pEF) (∗∗∗*p* < 0.001; ∗∗*p* < 0.01). DMSO, dimethyl sulfoxide; HA, hemagglutinin; HEK293T, human embryonic kidney 293T cell line; PSD95, postsynaptic density protein 95; RFP, red fluorescent protein; TTZ, tetrazole; zDHHC, zinc finger DHHC domain–containing protein.

PSD95 (postsynaptic density protein 95) is a major S-acylated protein that has a critical role in the maturation of excitatory neurons and in synaptic plasticity (46, 47). PSD95 can be S-acylated by zDHHC2, zDHHC3, zDHHC7, and zDHHC15 (6). Furthermore, PSD95 has been shown to be specifically S-acylated by zDHHC2 in response to synaptic activity blockade in primary hippocampal neurons (48). We therefore tested the effects of TTZ-1 and TTZ-2 on the S-acylation of PSD95 mediated by zDHHC2, zDHHC3, zDHHC7, and zDHHC15. Fig. 9 shows that, similar to the results obtained with GFP-SNAP25, a concentration of 25 μM of either TTZ-1 or TTZ-2 significantly inhibited PSD95-mCHERRY S-acylation by zDHHC2 and zDHHC15. In contrast, zDHHC3 and zDHHC7 activities toward PSD95 were unaffected by TTZ-1 and TTZ-2, mirroring the data obtained with GFP-SNAP25 as a substrate.

### Effect of TTZ-1 and TTZ-2 on the S-acylation of endogenous proteins in HEK293T cells

**Figure 10:**
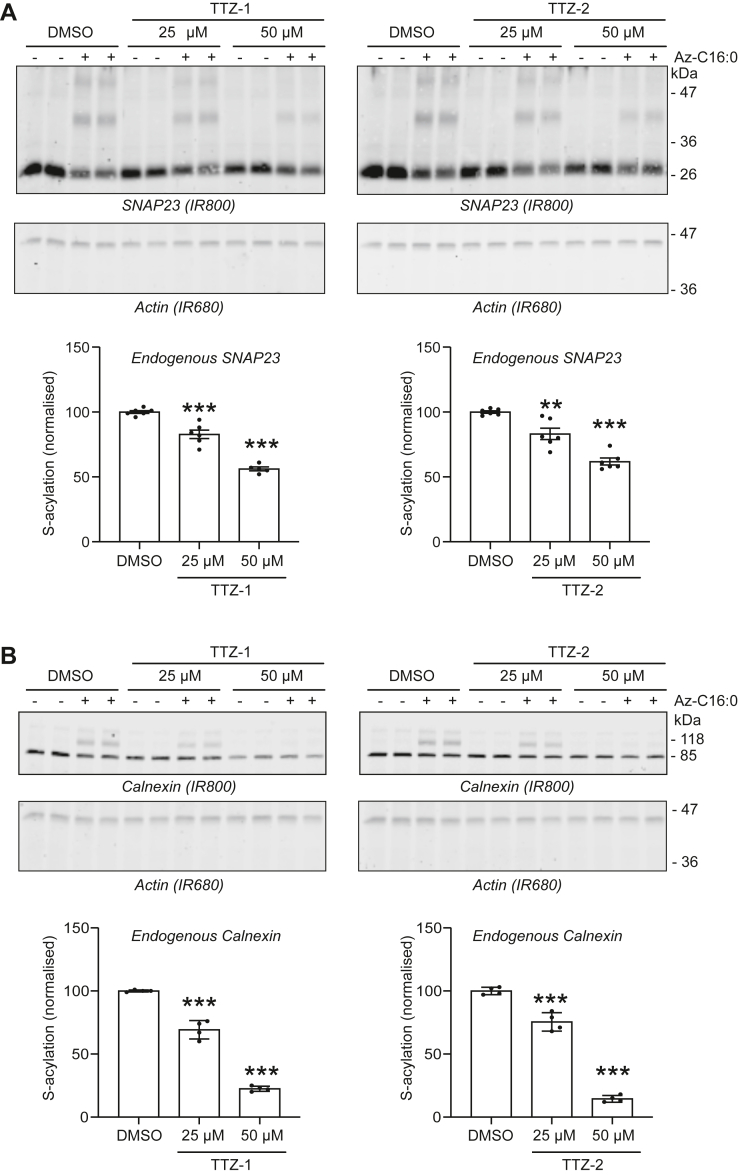
**Activity of TTZ-1 and TTZ-2 against the S-acylation of endogenous proteins in mammalian cells.** HEK293T cells were preincubated for 4 h with TTZ-1 or TTZ-2 (25 or 50 μM final) or DMSO control before metabolic labeling with 100 μM of either palmitic acid (−Az-C16:0) (as a control) or C16:0-azide (+Az-C16:0) for 4 h. Cells were lysed, and proteins having incorporated C16:0-azide were labeled by click chemistry with an alkyne-mPEG5K. Protein samples were then resolved by SDS-PAGE and transferred to nitrocellulose membranes that were first probed with either an anti-SNAP23 antibody (*A*) (IR800) or an anti-calnexin antibody (*B*) (IR800) and then probed with an anti-actin antibody (IR680). Representative images are shown. Positions of the molecular mass markers (in kilodalton) are indicated on the *right side* of the membranes. Two (*A*) or three (*B*) independent experiments were performed in duplicate. The data were quantified, and the graphs below each set of membranes show mean ± SEM of normalized S-acylation of either endogenous SNAP23 (*A*) or endogenous calnexin (*B*). *Filled circles* represent individual samples. Statistical analysis (ANOVA) was performed to reveal whether there was a significant difference between the substrate S-acylation of cells treated with DMSO *versus* similar samples treated with TTZ-1 or TTZ-2 (∗∗∗*p* < 0.001; ∗∗*p* < 0.01). DMSO, dimethyl sulfoxide; HEK293T, human embryonic kidney 293T cell line; TTZ, tetrazole.

Finally, we tested whether TTZ-1 and TTZ-2 were able to block the S-acylation of endogenous proteins in the absence of zDHHC enzyme overexpression. In order to detect the S-acylation of endogenous proteins, we used mPEG-click, a technique that we developed recently (49) that is better suited for the click chemistry–based analysis of S-acylation of endogenously expressed proteins. In this assay, cells are labeled with either palmitate (C16:0), or C16:0-azide, lysed, and then subjected to click chemistry with alkyne-mPEG 5K. The addition of mPEG 5K moieties to S-acylated proteins increases their apparent molecular weight, which can be detected directly by immunoblotting. HEK293T cells were pretreated for 4 h with either TTZ-1 or TTZ-2 at 25 and 50 μM and then labeled with fatty acids for another 4 h in the presence of the compounds. We first assessed the S-acylation of endogenous SNAP23 as it is a close relative of SNAP25 (SNAP25 is not expressed in the non-neuronal HEK293T cell line) (50, 51). Fig. 10*A* shows that SNAP23 S-acylation can indeed be detected by mPEG-click during the 4 h labeling period, as evidenced by the appearance of high molecular weight bands when cells are incubated with C16:0-azide but not with palmitate. The pretreatment of the cells with either TTZ-1 or TTZ-2 caused a significant reduction in SNAP23 S-acylation. Similarly, TTZ-1 and TTZ-2 decreased the S-acylation of calnexin, another well-characterized S-acylated protein (52, 53) (Fig. 10*B*).

## Discussion

Despite the increasing recognition of the importance of S-acylation in physiological and pathological processes, progress in understanding the widespread functions of this post-translational modification is slowed by a dearth of available chemical modulators for use as tool compounds. Very few assays that would be amenable for HTS have been developed for the detection of S-acylation in the past 10 years. An early assay was based on the fluorescent detection of the released free CoA following S-acylation (54). The subsequent screen of a scaffold ranking library consisting of 68 unique scaffolds and 30 million unique structures successfully identified several Erf2 inhibitors (41). A click chemistry–based HTS platform was also developed for the screening of Ras S-acylation inhibitors using membrane preparation as a source of enzyme, a biotinylated Ras peptide bound to streptavidin plates, and alkyne palmitoyl-CoA as a lipid substrate. S-acylation was detected by the addition of azido-CalFluor488, a probe that is nonfluorescent until it participates in a click reaction with an alkyne moiety (55). Although promising, no HTS results have yet been reported with the use of this technique. The most recent report (Acyl-cLIP) detects the change in fluorescence polarization of peptides (*i.e.*, substrates) that follows the post-translational attachment of lipid moieties and their subsequent binding to detergent micelles (56). A full HTS was thereafter performed to identify inhibitors of the activity of the *N*-palmitoyltransferase Hhat and will be reported in due course according to the authors. Acyl-cLIP was also tested for the detection of zDHHC3, zDHHC7, and zDHHC20 activities, showing that this assay can be adapted for the screening of zDHHC enzyme modulators (33, 39, 57).

Our focus here was to advance S-acylation research by (i) developing new assay platforms for inhibitor discovery and (ii) using these platforms to define novel chemical inhibitors of zDHHC enzymes. We showed that the developed high-throughput autoacylation assay was indeed a robust platform as it led to the identification of hits from a screen of >350,000 individual compounds that also inhibited enzyme autoacylation and substrate S-acylation in established cell-based assays. The compounds that emerged from this screen, TTZ-1 and TTZ-2, both possess a 2,8-disubstituted 3,4-dihydro-2H-benzo[b] [1,4]oxazine core. Common to both structures is a TTZ head group, a well-established bioisostere for carboxylic acids. In addition, both compounds contain a hydrophobic chain at the 8-position of the heterocycle linked through an amide bond. The resemblance of the polar and nonpolar components of these synthetic compounds to the natural fatty acid substrates for the zDHHC enzymes suggests that they may have a common binding site on the enzyme.

An important strength of the study is that the identified inhibitors displayed activity in multiple cell-free and cell-based assays, undertaken at different research sites. The compounds were indeed first identified for their inhibitory activity toward the autoacylation of zDHHC2, as measured by TR-FRET. TR-FRET is a popular choice for HTS campaigns because of its relative simplicity and has been widely used for the screening of modulators of molecular interactions (protein–protein or protein–DNA). TR-FRET has also been used to identify inhibitors of several enzymes, such as kinases, heparanases, and ubiquitin ligases (58). This is to our knowledge the first report of the adaptation of TR-FRET for the detection of protein lipidation, and one of the largest screens of compounds modulating zDHHC-mediated S-acylation. We have checked the specificity of the TR-FRET signal for S-acylation including using lipoparticles of inactive zDHHC2 mutant (C157S, Fig. 1*B*). We also attempted to develop a TR-FRET assay for substrate acylation. However, with the materials we used in this publication (NBD-tagged palmitoyl-CoA, HA-zDHHC2, and GST-SNAP25), we could not observe any TR-FRET signal on SNAP25 protein.

Following the identification of TTZ-1 and TTZ-2 in the HTS, the next step in the selection process was to screen their inhibitory activity toward substrate S-acylation in cell-free assays that used click chemistry for detection by either a newly developed ELISA or by direct SDS-PAGE analysis. A click-ELISA was developed previously for the detection of Hhat activity (59) with the use of streptavidin-coated plates for the attachment of a biotinylated substrate. S-acylation of the substrate was performed with purified enzyme and alkyne palmitoyl-CoA, which was then linked to a FLAG-tagged azide through copper-catalyzed click chemistry. A classic ELISA then followed for the detection of the FLAG epitope. In our hands, the standard click chemistry reagent mixture impaired the binding of the GST-tagged substrate to the glutathione plate, and we therefore had to implement a less widely used click chemistry technique. Copper-free click chemistry involves the biorthogonal reaction between DBCO and an azide moiety and is carried out in the physiological buffer PBS, which did not disrupt the efficient binding of GST to glutathione plate. Inconveniently, because of the reaction of DBCO with free cysteines, the (nonacylated) residues had to be blocked with NEM prior to the click chemistry reaction. It should be noted that, in addition to the main distinction that these different assays measured either autoacylation or substrate S-acylation, the TR-FRET and click chemistry assays also used different sources of zDHHC2 and different lipid substrates, highlighting the robustness of the generated data.

Finally, the compounds were tested for their ability to inhibit substrate S-acylation in cells overexpressing different zDHHC isoforms. Both inhibitors were active against the zDHHC2-mediated S-acylation of two different substrates, SNAP25 and PSD95. It is interesting to note that the inhibition profile of TTZ-1 and TTZ-2 on SNAP-25 S-acylation appeared to display some zDHHC isoform selectivity and was more potent against zDHHC2 and zDHHC15 *versus* zDHHC3 and zDHHC7, with zDHHC17 displaying intermediate levels of inhibition. Similarly, the inhibitors were more active against the S-acylation of PSD95 by zDHHC2 and zDHHC15 than by zDHHC3 and zDHHC7. We cautiously interpret these observations as indicating that the development of isoform-selective zDHHC inhibitors is a realistic possibility, perhaps because of subtle differences in the acyl-CoA binding pocket of zDHHC enzymes that affects lipid selectivity or differences in the turnover of the autoacylated enzyme intermediate; these possibilities will be explored in future work in this area. Fig. S3 shows a phylogram of the relatedness of the human zDHHC family members based on the amino acid sequence of the 51-amino acid DHHC-CRD. As can be seen, zDHHC2 and zDHHC15 are closely related, which is consistent with the strong inhibition of both enzymes by TTZ-1 and TTZ-2. In contrast, less inhibition was seen toward zDHHC3, zDHHC7, and zDHHC17, which are more distantly related to zDHHC2/zDHHC15. We note that zDHHC3 and zDHHC7 are highly active isoforms (60), and therefore, observed differences in inhibition profile might be related to differences, for example, in autoacylation turnover kinetics. To confirm isoform selectivity of these new S-acylation inhibitors will require detailed kinetic analyses using purified protein components, and therefore, we emphasize only the potential for these compounds to be further developed to enhance zDHHC enzyme isoform selectivity profiles. Nevertheless, the different effects of the compounds on different zDHHC enzymes does argue against their effects being indirect, for example, affecting fatty acid synthesis, uptake, or conversion to acyl-CoA.

The selective inhibition of zDHHC2-mediated S-acylation of PSD95 (and/or other substrates, such as AKAP79/150) in neurons could be facilitated by the use of TTZ-1 or TTZ-2 and might provide a therapeutic tool for the modulation of postsynaptic domain organization and of synaptic plasticity (48, 61, 62). zDHHC2 inhibition could also be of use for the management of several autoimmune disorders involving the lymphocyte-specific protein tyrosine kinase Lck. S-acylation of this protein, which is essential for T-cell activation, is mediated by zDHHC2 (63, 64, 65). The further development of more potent and selective inhibitors of zDHHC15 could have utility for the treatment of induced autoimmune disease involving the constitutive activation of STING (stimulator of interferon genes). STING can be S-acylated by zDHHC3, zDHHC7, and zDHHC15, and the inhibition of STING S-acylation effectively reduces its activity, perhaps offering opportunities for the treatment of some inflammatory diseases (66).

There may also be potential for employing selective inhibitors of zDHHC enzymes for the treatment of some infectious diseases. Many viral proteins undergo S-acylation, and this is often key for efficient viral replication (67, 68). Thus, acutely targeting the cellular enzymes that mediate S-acylation of essential viral proteins could interfere with viral infection. Indeed, the hemagglutinin of influenza A was recently shown to be modified by zDHHC2, zDHHC8, zDHHC15, and zDHHC20 (69). Similarly, the spike protein of SARS-CoV-2 can be S-acylated by zDHHC2, zDHHC3, zDHHC6, zDHHC9, zDHHC11, zDHHC12, zDHHC20, zDHHC21, and zDHHC24 (42, 70, 71), and siRNA-mediated knockdown of zDHHC20 impairs virus infectivity (70). Another relevant example is the report that zDHHC2 is one of the major enzymes (together with zDHHC19) responsible for S-acylation of the RNA-binding protein nsP1 of the alphavirus chikungunya. Disrupting nsP1 S-acylation severely impaired viral replication (72). The development of highly selective zDHHC inhibitors may therefore offer scope for blocking S-acylation of viral proteins, whilst limiting effects on the S-acylation of host proteins. Alternatively, targeting S-acylation of host proteins such as CCR5 has been suggested as a potential strategy for the inhibition of HIV-1 infection (73). Recent data suggest that zDHHC3, zDHHC7, and zDHHC15 are capable of S-acylating CCR5 when coexpressed in HEK293T cells, and that the inhibition of CCR5 S-acylation by two newly identified compounds is effective at reducing HIV-1 entry and replication in human macrophages. Interestingly, these compounds (cadmium chloride and zinc pyrithione) are both zinc chelators and proposed to act by binding directly to the zDHHC catalytic site of one or more of the enzymes identified; Zn^2+^ is known to bind to the DHHC-CRD of zDHHC enzymes and plays an important structural role.

As shown in Supplementary Fig. 3, zDHHC20 also clusters phylogenetically with zDHHC2/zDHHC15 (6, 20, 74), and it is therefore possible that the inhibitors identified here also target this enzyme. zDHHC20 has been implicated in the development of cancers resistant to epidermal growth factor receptor (EGFR) inhibitor therapies (75, 76, 77). Interestingly, zDHHC20 mediates EGFR S-acylation and inhibits EGF signaling; silencing of zDHHC20 restores EGFR sensitivity and sensitizes cells to EGFR inhibitor–induced cell death, indicating that inhibition of zDHHC20 activity could function therapeutically in combination with EGFR inhibitors (76). Blocking zDHHC20-mediated EGFR S-acylation has also been shown to reduce PI3K signaling and MYC levels and suppress cell growth *in vitro* and tumor growth in an *in vivo* model of KRAS-mutant lung adenocarcinoma (75).

Based on the results of the TR-FRET assays *in vitro* and of click chemistry in cell-based assays, the inhibitors described presumably target the first step of the enzymatic reaction, that is, the autoacylation of the enzyme, which makes them likely to inhibit the S-acylation of a broad range of substrates. Identifying inhibitors that are specific for a particular enzyme–substrate pair might be possible by targeting the interaction site between the two partners (30, 31, 32). With 23 human zDHHC enzymes and thousands of substrates, little is still known regarding the mode of interaction between enzyme and substrate (18, 32, 78). zDHHC3, zDHHC5, and zDHHC7 possess a PDZ-binding C-terminal domain allowing for the specific recruitment of some of their substrates (18, 78, 79, 80, 81). In contrast, other substrates seem to display a PDZ-independent mode of binding to zDHHC5 (82, 83), suggesting that compounds could be tailored to block specific enzyme–substrate pairs. For example, zDHHC5-mediated S-acylation of phospholemman, a small accessory subunit of the cardiac pump Na/K ATPase (NKA), is mediated through the recruitment of NKA by a juxtamembrane amphipathic α-helix of zDHHC5. Disrupting the interaction between NKA and zDHHC5 resulted in an inhibition of phospholemman S-acylation and was achieved by the addition of a specifically designed cell-penetrating peptide (84). There is also a growing understanding of how zDHHC17 recognizes and binds to its substrates. A key substrate recognition site between zDHHC17 and substrates is its N-terminal ankyrin repeat domain (ARD), which recognizes a conserved sequence known as the zDABM in its substrates (18, 85, 86, 87, 88, 89). Genistein has been recently identified as an inhibitor of MAP2K4 activity that disrupts the interaction between zDHHC17 ARD and MAP2K4, through direct binding to the ARD of zDHHC17; it would be worthwhile exploring the activity of genistein against other zDHHC17 substrates (90). A substrate-targeted approach has also been successful recently with the identification of ketoconazole as an inhibitor of the S-acylation and signaling of dual leucine-zipper kinase (91). Ketoconazole was also found to inhibit PSD95, but not GAP43, S-acylation, suggesting that this compound is not a general inhibitor of S-acylation and might have some zDHHC isoform selectivity. Although ketoconazole, and sulconazole (another compound identified in the study), both contain (similar to TTZ-1 and TTZ-2) an azole group, we do not believe that they act in a similar fashion. Indeed, ketoconazole contains an imidazole (Fig. S1), which is basic and would be expected to be protonated at physiological pH (*i.e.*, positively charged), whereas TTZ-1 and TTZ-2 contain a TTZ unit that is acidic and would be expected to be deprotonated at physiological pH (*i.e.*, negatively charged). We therefore expect ketoconazole and TTZ-1/TTZ-2 to have very different properties and hence biological targets/interactions.

The observation that TTZ-1/TTZ-2 inhibited the S-acylation of endogenous substrates mediated by endogenous enzymes is encouraging. Although the concentrations tested here were relatively high (25 and 50 μM), it has to be noted that the identity of the endogenous zDHHCs mediating the S-acylation of these substrates in HEK293T is unclear. Since these cells express the 23 members of the zDHHC family (91), a combination of several enzymes might be involved, including the more poorly inhibited zDHHC3/zDHHC7. It would be very interesting to test the effect of TTZ-1 and TTZ-2 in the regulation of synaptic activity, where zDHHC2 (but not zDHHC3) plays a crucial and specific role.

The identification of small-molecule inhibitors of zDHHC enzymes described herein provides valuable and much-needed tools to investigate S-acylation-dependent cellular pathways. Our data also suggest that they are capable of acting on native enzymes and substrates, highlighting their potential usefulness as tool compounds. It is envisaged that they will allow the acute effects of S-acylation disruption to be investigated without the need for long-term zDHHC depletion (*e.g.*, using RNAi or CRISPR technology). These tools are significantly more drug like in character and offer an important advance over nonselective inhibitors such as 2BP. In addition, the apparent selectivity of these compounds toward zDHHC2 and zDHHC15 offers the potential to refine and develop their selectivity further through a detailed structure–activity relationship study. Truly selective inhibitors of the zDHHC enzyme family would represent a major development for the field.

## Experimental procedures

### Cells

HEK293T cells (CRL-3216; American Type Culture Collection) were grown at 37 °C in a humidified atmosphere containing 5% CO_2_ in Dulbecco's modified Eagle's medium (DMEM) (catalog no.: 31966047; Gibco, Thermo Fisher Scientific) supplemented with 10% fetal bovine serum (catalog no.: 11550356; Gibco, Thermo Fisher Scientific). Insect cells Sf9 (catalog no.: 600100; Oxford Expression Technologies) and Sf9 TriEx (catalog no.: 71023-3; Novagen, Merck) were grown at 28 °C in a dry incubator without CO_2_, in ESF21 media (catalog no.: 500300; Oxford Expression Technologies) and TriEx medium (catalog no.: 71022; Novagen, Merck), respectively. All the components of the Expi293 MembranePro system (including the cells) were from Invitrogen.

### Antibodies

Goat GST antibody (catalog no.: 27457701V; GE Healthcare, used at 1:1000 dilution), NeutrAvidin-DyLight 800 (catalog no.: 22853; Invitrogen, used at 1:5000 dilution), and High-Sensitivity NeutrAvidin-HRP (catalog no.: 31030; Pierce, used at 1:20,000 dilution) were obtained from Thermo Fisher Scientific. Rat HA antibody (Roche; clone 3F10, used at 1:1000 dilution) was from Sigma, mouse GFP antibody (Clontech; clone JL8, used at 1:4000 dilution) was obtained from Takara, rabbit SNAP23 from Synaptic Systems (used at 1:1000 dilution), and mouse actin (clone AC40 A4700, used at 1:1000 dilution) was from Sigma. Sheep anti–red fluorescent protein antibody was a gift from Ian Prior (University of Liverpool). IR dye–conjugated secondary antibodies were used at a dilution of 1:20,000 and purchased from LI-COR Biosciences. Terbium cryptate–conjugated anti-HA antibody was from CisBio.

### DNA plasmids

Human complementary DNA (cDNA) encoding zDHHC2 (NM_016353.4), zDHHC3 (NM_001135179), zDHHC7 (NM_017740.2), zDHHC15 (NM_144969.2), and zDHHC17 (NM_015336.2) were synthesized by GeneArt Technologies (Thermo Fisher Scientific) and subcloned in pEF-BOS-HA (6) in frame with the triple HA tag coding sequence at the N-terminal end. GFP-SNAP25b plasmid has been described previously (92) and was originally a gift from Maurine Linder (Washington University School of Medicine, St Louis, MO). Rat PSD95 was subcloned in frame with a C-terminal mCHERRY tag in a pEGFP-N1 backbone where mCHERRY replaced the GFP coding sequence.

The human cDNA encoding zDHHC2 was codon optimized for protein expression in insect cells and synthesized by GeneArt Technology (Invitrogen). It was inserted with N- and C-terminal tags (His_6_ and StrepII, respectively) in the baculovirus transfer vector pIEX/Bac3 (Merck Novagen; catalog no.: 717263).

The validity of all constructs was verified by DNA sequencing (Dundee DNA Sequencing Service).

### Fatty acids

C16:0-azide has been described previously (13). Palmitoyl-CoA-NBD (catalog no.: 810705) was purchased from Avanti Polar Lipids. Azide-C16:0-CoA was synthesized using the method of Bishop and Hajra (93). Palmitic acid (PO500) was purchased from Sigma.

### HTS for the identification of zDHHC2 autoacylation inhibitors

The Expi293 MembranePro system was used to generate HA-tagged zDHHC2 lipoparticles according to the manufacturer’s instructions. Briefly, 90 ml of Expi293 cells were cotransfected with 30 μg of pEF-BOS-HA-zDHHC2 plasmid and 90 μg of a plasmid encoding the lentiviral protein Gag. A transfection enhancer was added the next day. The supernatant of the cell culture (containing the zDHHC2 lipoparticles) was collected 72 h post-transfection and mixed with the precipitation reagent provided in the kit for 1 or 2 days at 4°C. The lipoparticles were then pelleted by centrifugation at 6000*g* for 30 min at 4 °C and resuspended in the buffer provided. The optimized protocol for the HTS was as follows: compounds were tested at a final concentration on 4.2 μM (2.5 nl) premixed for a few minutes with 1.5 μl of HA-zDHHC2 lipoparticles (at 42 μg/ml) before the addition of 4.5 μl of a mixture of anti-HA Tb cryptate (1:200 dilution) and NBD-palmitoyl-CoA (1 μM). Compounds were dispensed by Echo 555 (Beckman Coulter), and other reagents were dispensed by Multi Drop Combi (Thermo Fisher Scientific). The TR-FRET signal was read after a 15 min incubation on a Biotek Synergy Neo apparatus (Agilent). The dynamic range during the campaign was between 1.5 and 2.3.

TR-FRET ratio was calculated by Biotek Gen5 software (Agilent) using the equation: acceptor signal/donor signal ∗ 10,000. The percent of inhibition was normalized by vehicle of WT enzyme (neutral control) and C157S inactive mutant (inhibitor control). Calculation of percent of inhibition and quality control of whole campaign was made with Screener software (Genedata).

One of the main strengths of this high-throughput assay is the absence of wash steps. Terbium homogenous TRF is indeed one of the only methods allowing for the detection of the interaction between tagged proteins and a probe without any wash. A potential limitation would be the distance between the FRET donor and the acceptor, which could be solved by testing other tags.

### Purification of recombinant proteins

The recombinant Baculovirus was obtained following the manufacturer’s instructions (BaculoComplete kit; Oxford Technologies; catalog no.: 400100). Briefly, Sf9 cells (Oxford Expression Technologies; catalog no.: 600100) were plated in 6-well plates and cotransfected with 1.2 μl of baculoFectin II, 100 ng of flashback Ultra DNA, and 500 ng of transfer vector (pIEX-Bac3-hDHHC2 or the control vector expressing lacZ). Cells were incubated for 5 days at 28 °C before the supernatant (containing the recombinant baculovirus seed stock) was harvested and stored at 4 °C until required. Virus amplification was performed in Sf9 TriEX cells (Novagen/Merck; catalog no.: 71023-3) grown in TriEx media (Novagen/Merck; catalog no.: 71022). The media were removed from cells in T75 flasks (at ∼50% confluence). About 100 μl of the seed stock virus was diluted to 2 ml with medium and added to the cells. The virus was allowed to adsorb for 1 h, with periodic rocking, before being removed. About 15 ml of fresh medium was then added, and the cells were incubated for at least 5 days before the medium containing recombinant virus was harvested (this virus stock is the inoculum) and centrifuged at 1000*g* for 20 min at 4 °C to remove broken cells. The recombinant viruses were titrated by serial dilution on 6-well plates of Sf9 cells and plaque titration.

Large-scale production of zDHHC2 was performed in Sf9 TriEX cells. Medium was removed from 15 T75 flasks of TriEX cells, and the recombinant virus was added to the cells in a total volume of 2 ml and at a multiplicity of infection of 2. The virus was allowed to adsorb at room temperature for 1 h, during which time the cells were occasionally gently rocked. The inoculum was then removed and replaced with 13 ml of fresh TriEX medium and incubated for 96 h at 28 °C. The cells were then detached by tapping the flask and collected by centrifugation at 1000*g* for 5 min. About 10 ml of PBS (pH 6.4) was added to the flask to collect the remaining cells, which were combined with the cell pellets and centrifuged again at 1000*g* for 5 min. The cell pellets were then washed two further times in PBS (pH 6.4) and resuspended in a small volume of PBS supplemented with protease inhibitors (50 × stock contains 800 μg/ml benzamidine HCl [Sigma; catalog no.: B65506], 500 μg/ml aprotinin [Sigma; catalog no.: A1153], 500 μg/ml leupeptin [Sigma; catalog no.: L2884], 500 μg/ml pepstatin A [Sigma; catalog no.: P5318], and 50 mM PMSF [Sigma; catalog no.: P7626]), pelleted, and frozen at −80 °C until required.

Cell pellets were thawed and resuspended in lysis buffer (50 mM Tris [Sigma; catalog no.: T6066], pH 7.4, 200 mM NaCl [Sigma; catalog no.: 31434], 10% glycerol [Fisher; catalog no.: G/0600/17], 1% *n*-dodecyl-β-d-maltoside [DDM; Thermo; catalog no.: 89903], 1 mM Tris(2 carboxyethyl) phosphine hydrochloride [TCEP; Sigma; catalog no.: C4706], and protease inhibitors [as aforementioned]). About 1 ml of lysis buffer was added per cell pellet (from three T75 flasks), and the cells were disrupted further by 10 strokes of a Dounce homogenizer. The cell lysates were then rotated for 30 min at 4 °C on a wheel at a gentle speed and then clarified by ultracentrifugation at 100,000*g* for 30 min at 4 °C. The lysates were then supplemented with 5 to 10 mM imidazole (Sigma; catalog no.: I5513), added to 1 ml of washed Ni^2+^–nitrilotriacetic acid agarose (Qiagen; catalog no.: 1018244), and rotated on a low-speed wheel for at least 1 h at 4 °C. The agarose was then pelleted by centrifugation at 2000*g* for 5 min at 4 ^°^C and washed five times with 10 ml of wash buffer (50 mM Tris [pH 7.4], 200 mM NaCl, 10% glycerol 0.2% DDM, and 0.5 mM TCEP) containing 15 mM imidazole. The washed agarose beads were resuspended in 500 μl of elution buffer 1 (50 mM Tris [pH 7.4], 100 mM NaCl, 10% glycerol, 0.1% DDM, 0.25 mM TCEP, and 200 mM imidazole) and rotated on a wheel for 10 min at 4 °C. The agarose was then pelleted by centrifugation at 2000*g* for 5 min at 4 °C. This elution step was repeated once. The agarose beads were then eluted four times with 500 μl of elution buffer 2 containing 500 mM imidazole (50 mM Tris [pH 7.4], 100 mM NaCl, 10% glycerol, 0.1% DDM, 0.25 mM TCEP, and 500 mM imidazole). The eluted samples were pooled (∼3 ml) and dialyzed twice (overnight and for 4 h) against 500 ml of elution buffer without imidazole or TCEP. Purified proteins were analyzed by SDS-PAGE and Instant Blue (catalog no.: 10616474; Expedeon, Thermo Fisher Scientific) staining. The concentration of the enzyme was determined by comparison with bovine serum albumin (BSA) standards and found to be between 1.5 and 3 μM depending on the batch.

The production and purification of GST and GST-SNAP25b has been described (87). Briefly, *Escherichia coli* BL21(DE3)pLysS cells (catalog no.: L1195; Promega) were transformed with either pGEX-KG (containing the GST cDNA) or pGEX-KG-rat SNAP25b (for the production of SNAP25b with an N-terminal GST tag) and selected with the appropriate antibiotics. Transformed cells were grown in a 1 l culture of supermedia (150 mM NaCl, 1.5% tryptone [Oxoid; catalog no.: LP0043], 2.5% yeast extract [Oxoid; catalog no.: LP0021]) with shaking at 225 rpm (37 °C), and protein expression was induced by the addition of 1 mM IPTG for 5 to 6 h. Cells were pelleted and resuspended in PBS. The cells were lysed by subjecting to a freeze–thaw cycle at −80 °C, addition of 1 mg/ml lysozyme (Fluka; catalog no.: 62971) and incubation for 30 min on ice. The cells were then sonicated, and cell debris and membranes removed by centrifugation at 20,000*g* for 60 min. The clarified lysates were incubated with glutathione sepharose (1 ml bed volume; GE Healthcare; catalog no.: 17-0756-01), washed, and eluted by the addition of 2 × 1.5 ml of 10 mM reduced l-glutathione (Sigma; #catalog no.: T6066) in 50 mM Tris (pH 8). Eluted proteins were dialyzed overnight at 4 °C against 5 l of PBS in a 3.5 molecular weight cutoff slide-A-lyzer G2 cassette (catalog no.: 87723; Thermo). Purified proteins were analyzed by SDS-PAGE and Instant Blue staining. Their concentration was estimated from the intensity of their corresponding bands as compared with the standard curve obtained with BSA standards that were run in parallel.

### *In vitro* substrate S-acylation followed by copper-free click chemistry

In an uncoated 96-well plate, 0.075 μM of purified zDHHC2 enzyme was incubated with 1 μM GST-tagged substrate protein and 1 μM azide-C16:0-CoA at 25 °C for 45 min. The assay buffer consisted of 50 mM MES (pH 6.4), 100 mM NaCl, 0.1% DDM, and 1 mM TCEP. Following the reaction, NEM was added to a final concentration of 50 mM, and the plate was incubated for 2 h at room temperature with gentle rocking. Following this, 10 μl of click reagent (dibenzocyclooctyne biotin conjugate; Sigma; catalog no.: 760749) diluted in PBS was added (5 μM final) and incubated at room temperature for 1 h with rocking. About 100 μl of PBS was added to each well of the uncoated plate to dilute the samples. For assessment of ONO compounds using this assay, the compounds were preincubated with enzyme for 15 min at room temperature prior to the addition of substrates. The concentration of compounds used refers to their final concentration in the S-acylation reaction, and they were therefore added at a higher initial concentration (1.66×) for the 15 min preincubation period.

### Detection of *in vitro* substrate S-acylation by SDS-PAGE and Western blotting

Samples prepared as detailed previously were supplemented with 4 × SDS-PAGE loading buffer (containing 100 mM DTT). They were heated at 95 °C for 5 min and loaded on an SDS-PAGE gel before being transferred to a nitrocellulose membrane. The membrane was incubated with NeutrAvidin-800 and a goat anti-GST antibody and then an anti–goat-680 antibody before being imaged on a LI-COR Odyssey infrared scanner.

### Measurement of *in vitro* substrate S-acylation by ELISA

Glutathione-coated plates were activated (according to the manufacturer's instructions; Pierce glutathione-coated black 96-well plates [Thermo; catalog no.: 15340]) with two brief washes with 200 μl of PBS 0.05% v/v Tween-20. About 80 μl of PBS was then added to each well, and 20 μl of each sample (prepared as aforementioned) was added (in duplicate) and incubated at room temperature for 1 to 2 h on a rocking platform. The plate was then washed several times with 200 μl of PBS-0.05% Tween-20. About 100 μl of NeutrAvidin-HRP (Pierce High Sensitivity NeutrAvidin HRP, Thermo Fisher Scientific; catalog no.: 31030) diluted at 1:20,000 in PBS 0.025% Tween-20 was added to each well and incubated at room temperature for 30 min on a rocking platform. The plate was then washed several times with 250 μl of PBS-0.05% Tween-20 and incubated with 100 μl of SuperSignal ELISA Pico chemiluminescent substrate (Thermo Fisher Scientific; catalog no.: 37069). The resulting signal was read 10 min post addition of the substrate. For quantification, the average value of GST-SNAP25 samples (without zDHHC2) was subtracted from all raw values. These values were then normalized to the value of the DMSO control sample containing GST-SNAP25 and zDHHC2, providing a percentage of activity remaining after treatment with inhibitors. The percentage inhibition was then calculated as (100 − percentage activity remaining). Data were analyzed and fitted to a nonlinear curve using the log(inhibitor) *versus* normalized response (variable slope) equation (GraphPad Software).

### Assessment of the activity of compounds on S-acylation in mammalian cells

HEK293T cells were plated on poly-d-lysine–coated 24-well plates (catalog no.: 356413; Corning BioCoat, VWR) and transfected with 2.4 μg of pEF-zDHHC2 plasmid (or the control empty plasmid pEF-BOS-HA) for the measurement of zDHHC2 autoacylation and 4.8 μl of Lipofectamine 2000 (Invitrogen). For substrate acylation, cells were transfected with 1.6 μg of pEF-zDHHC plasmid (or the control empty plasmid pEF-BOS-HA) together with 0.8 μg of EGFP-SNAP25b plasmid and 4.8 μl of Lipofectamine 2000. Twenty-four hours post-transfection, cells were washed once with PBS and preincubated with ONO or UoS compounds diluted in 250 μl of serum-free DMEM for 4 h. S-acylation labeling was then started by the addition of 50 μl of 600 μM of either unmodified palmitic acid C16:0 or C16:0-azide (final concentration of 100 μM) diluted in serum-free DMEM supplemented with 1 mg/ml fatty acid–free BSA (Sigma; catalog no.: A7030). Cells were labeled for 4 h, washed once with PBS, then lysed on ice in 100 μl of 50 mM Tris (pH 8) containing 0.5% SDS and protease inhibitors (Sigma; catalog no.: P8340). Conjugation of IR800 alkyne dye or mPEG5K alkyne to C16:0-azide was carried out for 1 h at room temperature with end-over-end rotation by adding an equal volume of freshly prepared click chemistry reaction mixture containing the following: 5 μM IRDye 800CW alkyne (catalog no.: 929-60002; Li-COR) or 200 μM of mPEG5K-alkyne (catalog no.: JKA3177; Sigma), 4 mM CuSO_4_ (catalog no.: 451657; Sigma), 400 μM Tris[(1-benzyl-1*H*-1,2,3-triazol-4-yl)methyl]amine (catalog no.: 678937; Sigma), and 8 mM ascorbic acid (catalog no.: A15613; Alpha Aesar) in distilled water. About 67 μl of 4 × SDS-PAGE sample buffer containing 100 mM DTT was then added to the 200 μl sample. Samples were heated at 95 °C for 5 min, and 20 μl was resolved by SDS-PAGE and transferred to nitrocellulose membrane for immunoblot analysis. Membranes were first stained with Revert 700 total protein stain kit (catalog no.: 926-11016; LI-COR), imaged, destained, and then analyzed by immunoblotting. Signals were quantified with ImageStudio software (LI-COR). S-acylation was calculated as the ratio (in percent) between click signal (IR800) and GFP signal (IR680) and normalized to control conditions on the same membrane (cells expressing GFP-SNAP25 + zDHHC and treated with DMSO). For alkyne mPEG-treated samples, the efficiency of S-acylation was calculated as the ratio between the sum of the signals of the higher molecular weight bands (corresponding to modified cysteines) over the sum of all the signals. The expression of the various zDHHCs was also quantified as a ratio between the HA signal (IR680) and the Total Protein Stain (IR680) and normalized to control conditions on the same membrane (cells expressing GFP-SNAP25 + zDHHC and treated with DMSO). For the determination of the in cell IC_50_, the percent of S-acylation inhibition was calculated as (100 − percent of S-acylation). Data were analyzed and fitted to a nonlinear curve using the log(inhibitor) *versus* normalized response (variable slope) equation (GraphPad Software).

### Phylogenic analysis of ZDHHC

The following human ZDHHC protein sequences were retrieved from the National Center for Biotechnology Information database: ZDHHC1 (NP_001310556.1), ZDHHC2 (NP_057437.1), ZDHHC3 (NP_001128651.1), ZDHHC4 (NP_001358225.1), ZDHHC5 (NP_056272.2), ZDDHC6 (NP_071939.1), ZDHHC7 (NP_060210.2), ZDHHC8 (NP_037505.1), ZDHHC9 (NP_057116.2), ZDHHC11 (NP_079062.1), ZDHHC12 (NP_001304944.2), ZDHHC13 (NP_061901.2), ZDHHC14 (NP_078906.2), ZDHHC15 (NP_659406.1), ZDHHC16 (NP_115703.2), ZDHHC17 (NP_056151.2), ZDHHC18 (NP_115659.1), ZDHHC19 (NP_001034706.1), ZDHHC20 (NP_694983.2), ZDHHC21 (NP_001341047.1), ZDHHC22 (NP_777636.2), ZDHHC23 (NP_001307395.1), and ZDHHC24 (NP_997223.1). Sequences corresponding to the 51 amino-acid DHHC CRD were aligned with MUSCLE, phylogenic analysis was performed with PhyML, and the tree was rendered with TreeDyn. They were analyzed on www.phylogeny.fr.

### Synthesis of TTZ-1 and TTZ-2

#### 2,6-Dinitroaniline (6)

Chlorobenzene **3** (20.0 ml, 0.20 mol) was added to concentrated sulfuric acid (150 ml), and the solution was heated at 120 °C for 4 h (94). After cooling the reaction to room temperature, KNO_3_ (68.0 g, 0.6 mol) was added in four portions while stirring. The reaction was then heated at 115 °C for 20 h while stirring. While still hot, the solution was poured onto 1 kg of ice, once all the ice was melted the suspension was filtered and pressed dry. The solid was then dissolved in hot distilled water (240 ml), and most of insoluble solids were removed by decantation and filtration of the solution while still hot. The product was then left to crystallize and filtered to give a pale-yellow solid that was dissolved in aqueous ammonia (180 ml) and water (180 ml). The mixture was left stirring at reflux for 1 h. The solution was cooled down to 4 °C for 12 h, and the solid was filtered under suction to give an orange solid that was dissolved in 100 ml of concentrated sulfuric acid and 100 ml of water. The solution was heated at reflux for 16 h. While hot, the solution was poured onto 1 kg of cracked ice. When the ice had melted, the solid was filtered under vacuum to give the *title compound* **6** as a yellow solid (12.9 g, 36%). Melting point (mp): 138 °C (literature value [Lit]: 137–142 °C) (94); low-resolution mass spectrometry (LRMS) (electrospray ionization [ESI] + atmospheric pressure chemical ionization [APCI]): *m/z* calculated 183.1, found 182.1 [M − H]^−^; υ_max_ (thin film, cm^−1^) 3462, 3345, 3107, 1636, 1513; ^1^H NMR (400 MHz, DMSO-*d*_6_) δ 8.49 (d, *J* = 8.3 Hz, 2H), 8.39 (s, 2H), 6.84 (t, *J* = 8.3 Hz, 1H); ^13^C NMR (101 MHz, DMSO-*d*_6_) δ 140.8, 134.9, 134.0, 113.8.

#### 2-Chloro-1,3-dinitrobenzene (7)

Sodium nitrite (1.09 g, 15.8 mmol) was added to concentrated sulfuric acid (11 ml), and the resulting solution was heated at 40 °C for 20 min to ensure that all the solid was dissolved, then a solution of 2,6-dinitroaniline **6** (2.63 g, 14.4 mmol) in hot acetic acid (27 ml) was added at such a rate that the temperature was kept under 40 °C (95). After the addition was complete, the reaction was heated at 40 °C for 1 h. Copper(I) chloride (2.1 g, 21.2 mmol) was added to ice-cold hydrochloric acid (27 ml), and then the diazonium salt solution was added so that the effervescence was kept under control. After the addition was finished, the reaction was heated at 80 °C until the effervescence stopped. The reaction mixture was cooled to 4 °C for 12 h, and then the solid was filtered under vacuum to give the *title compound* **7** as a yellow solid (1.65 g, 57%). mp: 87 °C (Lit: 86–87 °C) (95); υ_max_ (thin film, cm^−1^) 3103, 1701, 1528; ^1^H NMR (400 MHz, acetone-*d*_6_) δ 8.33 (d, *J* = 8.2 Hz, 2H), 7.93 (t, *J* = 8.2 Hz, 1H), 6.01 (bs, 1H); ^13^C NMR (101 MHz, acetone-*d*_6_) δ 150.5, 130.6, 129.3, 119.7.

#### 2,6-Dinitrophenol (8)

2-Chloro-1,3-dinitrobenzene **7** (1.65 g, 8.14 mmol) and potassium hydroxide (1.37 g, 16.3 mmol) were added to water (30 ml), and the solution was heated to reflux for 1 h (96). The hot solution was added to ice-cold water (30 ml), and the pH was adjusted to 5 through the addition of concentrated HCl. The solution was extracted with EtOAc (25 ml, three times), the combined organic phase was dried over anhydrous magnesium sulfate, and the solvent was removed under reduced pressure to give the *title compound* **8** as a red solid (1.50 g, quant). mp: 63 °C (Lit: 63.5 °C) (96); LRMS (ESI + APCI): *m/z* calculated 184.1, found 183.1 [M − H]^−^; υ_max_ (thin film, cm^−1^) 3215, 3096, 1767, 1932, 1809, 1692, 1623, 1534; ^1^H NMR (400 MHz, acetone-*d*_6_) δ 8.35 (d, *J* = 8.3 Hz, 2H), 7.20 (t, *J* = 8.3 Hz, 1H); ^13^C NMR (101 MHz, acetone-*d*_6_) δ 150.3, 139.8, 131.8, 118.8.

#### 2-Amino-6-nitrophenol (9)

Hydrogen was bubbled through a nitrogen purged solution of 2,6-dinitrophenol **8** (1.5 g, 8.15 mmol) and 10% palladium on activated carbon (150 mg, 1.60 mmol) in methanol (20 ml) for 15 min (97). The reaction mixture was then left stirring under a hydrogen atmosphere for 45 min. The reaction was monitored by TLC. After the starting material was consumed, the reaction was filtered through Celite and eluted with acetone (50 ml) and the solvent was removed *in vacuo*. The crude was purified by flash chromatography (toluene:dichloromethane 9:1) to give the *title compound* **9** as a red solid (1.1 g, 88%). mp: 110 °C; LRMS (ESI + APCI): *m/z* calculated 154.1, found 155.1 [M + H]^+^; υ_max_ (thin film, cm^−1^) 3481, 3385, 3223, 3103, 2921, 2852, 1538; ^1^H NMR (500 MHz, chloroform-d) δ 10.73 (s, 1H), 7.48 (dd, *J* = 8.6, 1.5 Hz, 1H), 6.95 (dd, *J* = 7.7, 1.5 Hz, 1H), 6.79 (dd, *J* = 8.6, 7.7 Hz, 1H), 4.09 (s, 2H); ^13^C NMR (101 MHz, chloroform-d) δ 143.4, 137.9, 133.8, 120.5, 119.9, 113.6.

#### 8-Nitro-3,4-dihydro-2*H*-benzo[*b*][1,4]oxazine-2-carbonitrile (11)

2-Amino-6-nitrophenol **9** (500 mg, 3.24 mmol) and cesium carbonate (2.11 g, 6.48 mmol) were dissolved in toluene (18 ml) and heated at 50 °C for 30 min. Then, 2-chloroacrylonitrile **10** (2.0 ml, 25.95 mmol) was added, and the reaction was heated at 110 °C in a sealed vial for 4 h. After the reaction was completed, the solvent was removed under reduced pressure to give a brown solid, which was filtered through a Celite plug and eluted with acetone. The solvent was removed *in vacuo* to give a brown solid. The crude product was purified on silica gel (toluene:EtOAc 8:2) to give the *title compound* **11** as a brown solid (100 mg, 15%). LRMS (ESI + APCI): *m/z* calculated 205.2, found 204.0 [M − H]^−^; υ_max_ (thin film, cm^−1^) 3392, 3111, 2927, 1780, 1688, 1616, 1532; ^1^H NMR (500 MHz, acetone-d6) δ 7.17 (dd, *J* = 8.1, 1.7 Hz, 1H), 7.05 (dd, *J* = 8.1, 1.7 Hz, 1H), 6.99 (at, *J* = 8.1 Hz, 1H), 6.15 (bs, 1H), 5.63-5.60 (m, 1H), 3.89-3.75 (m, 2H); ^13^C NMR (101 MHz, acetone) δ 141.2, 136.1, 134.3, 123.1, 120.0, 116.8, 114.1, 63.2, 43.0.

#### 8-Amino-3,4-dihydro-2*H*-benzo[*b*][1,4]oxazine-2-carbonitrile (12)

8-Nitro-3,4-dihydro-2*H*-benzo[*b*][1,4]oxazine-2-carbonitrile **11** (50 mg, 0.24 mmol) and 10% palladium on activated carbon (5 mg, 10 mol%) were dissolved in methanol (5 ml), and the solution was purged with hydrogen for 15 min and left stirring under a hydrogen atmosphere for 45 min. The solution was filtered through Celite, eluted with acetone, and the solvent was removed under vacuum to give the *title compound* **12** as a brown oil (29 mg, 70%). LRMS (ESI + APCI): *m/z* calculated 175.2, found 176.1 [M + H]^+^; υ_max_ (thin film, cm^−1^) 3370, 2966, 2863, 2251, 1623, 1498; ^1^H NMR (400 MHz, chloroform-d) δ 6.68 (at, *J* = 8.0 Hz, 1H), 6.22 (dd, *J* = 8.0, 1.4 Hz, 1H), 6.12 (dd, *J* = 8.0, 1.4 Hz, 1H), 5.15-5.10 (m, 1H), 3.85 (bs, 1H), 3.74 (bs, 2H), 3.65-3.60 (m, 2H);^13^C NMR (101 MHz, CDCl_3_) δ 136.3, 132.0, 129.2, 123.1, 116.4, 107.2, 106.5, 62.7, 43.9.

#### *N*-(2-Cyano-3,4-dihydro-2*H*-benzo[*b*][1,4]oxazin-8-yl)-4-(heptyloxy)benzamide (15)

4-(Heptyloxy)benzoic acid **13** (5 mg, 0.02 mmol) and HATU (8 mg, 0.02 mmol) were dissolved in DMF (1 ml) and *N,N*-diisopropylethylamine (11 μl, 0.06 mmol) was added, and the resulting solution was left stirring for 45 min. A solution of compound **12** (4 mg, 0.02 mmol) in DMF (100 μl) was added to the acid solution, and it was left stirring at room temperature for 16 h. An aqueous solution of 5% lithium chloride (30 ml) was added, and the aqueous phase was extracted with EtOAc (20 ml, three times), the organic fractions were combined and dried over anhydrous MgSO_4_, the organic solution was filtered, and the solvent was removed by rotatory evaporation. The crude was purified by flash chromatography (PhMe:EtOAc 8:2) to give the *title compound* **15** as a brown oil (6 mg, 58%). LRMS (ESI + APCI): *m/z* calculated 393.2, found 394.2 [M + H]^+^; υ_max_ (thin film, cm^−1^) 3381, 2956, 2932, 2859, 2552, 1662, 1610, 1506;^1^H NMR (400 MHz, acetone-d6) δ 8.68 (bs, 1H), 7.96-7.92 (m, 2H), 7.71 (dd, J = 8.1, 1.5 Hz, 1H), 7.09-7.01 (m, 2H), 6.82 (at, J = 8.1 Hz, 1H), 6.53 (dd, J = 8.1, 1.5 Hz, 1H), 5.58-5.51 (m, 2H), 4.09 (t, J = 6.5 Hz, 2H), 3.79-3.65 (m, 2H), 1.86-1.76 (m, 2H), 1.55-1.26 (m, 8H), 0.96-0.83 (m, 3H); ^13^C NMR (101 MHz, acetone) δ 165.0, 163.0, 134.0, 131.6, 130.0, 129.2, 128.1, 123.0, 117.5, 115.1, 111.8, 68.9, 63.5, 43.9, 32.6, 26.7, 23.3, 14.3; three carbons missing.

#### *N*-(2-Cyano-3,4-dihydro-2*H*-benzo[*b*][1,4]oxazin-8-yl)-4-(4-phenylbutoxy)benzamide (16)

4-(Benzyloxy)benzoic acid **14** (6 mg, 0.02 mmol) and HATU (8 mg, 0.21 mmol) were dissolved in DMF (1 ml) and *N,N*-diisopropylethylamine (11 μl, 0.06 mmol) was added, and the resulting solution was left stirring for 45 min. A solution of compound **12** (4 mg, 0.02 mmol) in DMF (100 μl) was added to the acid solution, and it was left stirring at room temperature for 16 h. A solution of 5% lithium chloride (30 ml) was added, and the aqueous phase was extracted with EtOAc (15 ml, three times), the organic fractions were combined and dried over anhydrous MgSO_4_, the organic solution was filtered, and the solvent was removed by rotatory evaporation. The crude was purified by flash chromatography (PhMe:EtOAc 8:2) to give the *title compound* **16** as a brown oil (4 mg, 45%). LRMS (ESI + APCI): *m/z* calculated 427.2, found [M + H]^+^; υ_max_ (thin film, cm^−1^) 3375, 3087, 3062, 3029, 2940, 2863, 2543, 1660, 1608; ^1^H NMR (400 MHz, acetone-d6) δ 8.68 (bs, 1H), 7.97-7.91 (m, 2H), 7.72 (dd, J = 8.1, 1.5 Hz, 1H), 7.32-7.22 (m, 4H), 7.20-7.14 (m, 1H), 7.06-7.01 (m, 2H), 6.82 (at, J = 8.1 Hz, 1H), 6.53 (dd, J = 8.1, 1.5 Hz, 1H), 5.55-5.51 (m, 2H), 4.15-4.09 (m, 2H), 3.77-3.64 (m, 2H), 2.71 (t, J = 7.0 Hz, 2H), 1.91-1.72 (m, 4H); ^13^C NMR (101 MHz, acetone) δ 165.0, 162.9, 143.2, 134.0, 131.6, 130.0, 129.2, 129.1, 128.1, 126.6, 123.0, 117.5, 115.1, 111.9, 68.7, 63.5, 43.9, 36.1, 28.7; three carbons missing.

#### *N*-(2-(1H-Tetrazol-5-yl)-3,4-dihydro-2H-benzo[b][1,4]oxazin-8-yl)-4-(heptyloxy)benzamide (TTZ-1) (1)

Compound **15** (6 mg, 0.018 mmol) was dissolved in DMF (100 μl) and then sodium azide (4 mg, 0.056 mmol) and triethylammonium chloride (8 mg, 0.056 mmol) were added and the vial was sealed and heated at 140 °C for 2 h. The contents of the vial were left to cool down to room temperature, and a solution of 5% lithium chloride in water (30 ml) was added, the aqueous phase was extracted with ether (15 ml, three times), the organic fractions were combined, and the solvent was removed *in vacuo* to give the *title compound* TTZ-1 (**1**) as a brown oil (7 mg, quant). LRMS (ESI + APCI): *m/z* calculated 436.2, found 435.1 [M − H]^−^; υ_max_ (thin film, cm^−1^) 3325, 3271, 2951, 2928, 2874, 2859, 2735, 2617, 2470, 1640; ^1^H NMR (400 MHz, acetone-d6) δ 9.49 (bs, 1H), 8.11-8.04 (m, 2H), 7.12-7.03 (m, 2H), 6.84 (dd, J = 8.0, 1.5 Hz, 1H), 6.75 (at, J = 8.0 Hz, 1H), 6.52 (dd, J = 8.0, 1.5 Hz, 1H), 5.89 (at, J = 3.0 Hz, 1H), 5.43 (bs, 1H), 4.11 (t, J = 6.5 Hz, 2H), 4.03-3.78 (m, 2H), 1.90-1.74 (m, 2H), 1.53-1.45 (m, 2H).1.59-1.20 (m, 6H), 0.95-0.82 (m, 3H); ^13^C NMR (101 MHz, acetone-d6) δ 167.5, 163.4, 156.7, 136.1, 135.2, 130.7, 127.1, 126.7, 122.6, 115.7, 115.2, 114.3, 69.2, 69.0, 43.5, 32.5, 29.9, 26.7, 23.3, 14.3; 1 carbon missing.

#### *N*-(2-(1*H*-Tetrazol-5-yl)-3,4-dihydro-2*H*-benzo[*b*][1,4]oxazin-8-yl)-4-(4-phenylbutoxy)benzamide (TTZ-2) (2)

Compound **16** (4 mg, 0.011 mmol) was dissolved in DMF (100 μl), and then sodium azide (3 mg, 0.046 mmol) and triethylammonium chloride (3 mg, 0.022 mmol) were added and the vial was sealed and heated at 140 °C for 2 h. The contents of the vial were left to cool down to room temperature, and a solution of 5% lithium chloride in water (30 ml) was added, the aqueous phase was extracted with ether (15 ml, three times), the organic fractions were combined, and the solvent was removed *in vacuo* to give the *title compound* TTZ-2 (**2**) as a brown oil (5 mg, quant). LRMS (ESI + APCI): *m/z* calculated 470.2, found 469.1 [M − H]^−^; υ_max_ (thin film, cm^−1^) 3331, 3027, 2928, 2858, 1701, 1638, 1608; ^1^H NMR (400 MHz, acetone-d6) δ 9.49 b(s, 1H), 8.11-8.04 (m, 2H), 7.32-7.14 (m, 5H), 7.11-7.04 (m, 2H), 6.84 (dd, J = 8.0, 1.6 Hz, 1H), 6.75 (at, J = 8.0 Hz, 1H), 6.52 (dd, J = 8.0, 1.6 Hz, 1H), 5.89 (at, J = 3.0 Hz, 1H), 5.43 (bs, 1H), 4.21-4.07 (m, 2H), 4.03-3.74 (m, 2H), 2.75-2.68 (m, 2H), 1.89-1.77 (m, 4H); ^13^C NMR (101 MHz, acetone-d6) δ 168.1, 168.0, 167.5, 163.4, 151.8, 143.2, 130.7, 129.3, 129.2, 126.6, 122.5, 115.7, 115.2, 114.3, 69.2, 68.8, 55.5, 43.5, 36.1, 28.7; two carbons missing.

## Data availability

All data are contained within the article. Copies of ^1^H NMR and ^13^C NMR spectra are available as supporting information.

## Supporting information

This article contains supporting information.

## Conflict of interest

The authors declare that they have no conflicts of interest with the contents of this article.
