# Supplementary material for: Development of a novel high-throughput screen for the identification of new inhibitors of protein S-acylation
Source: J Biol Chem. 2022 Sep 8;298(10):102469. doi: 10.1016/j.jbc.2022.102469 (PMC9558053; doi:10.1016/j.jbc.2022.102469)

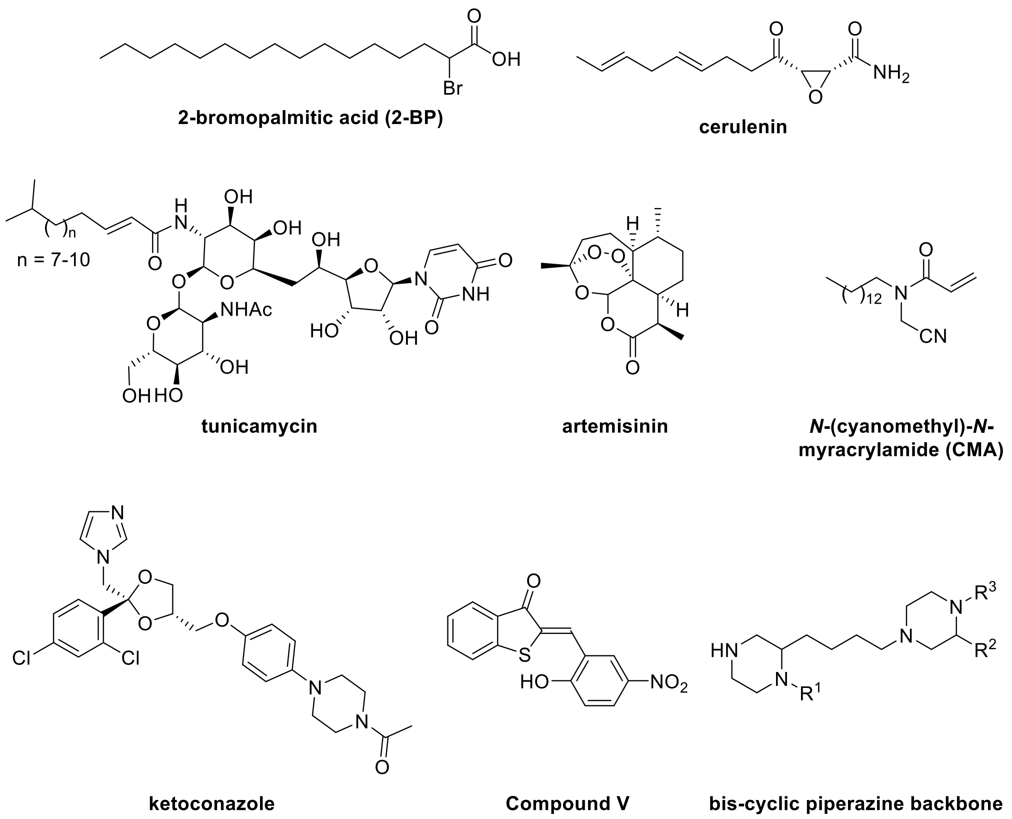

Supplementary Figure 1

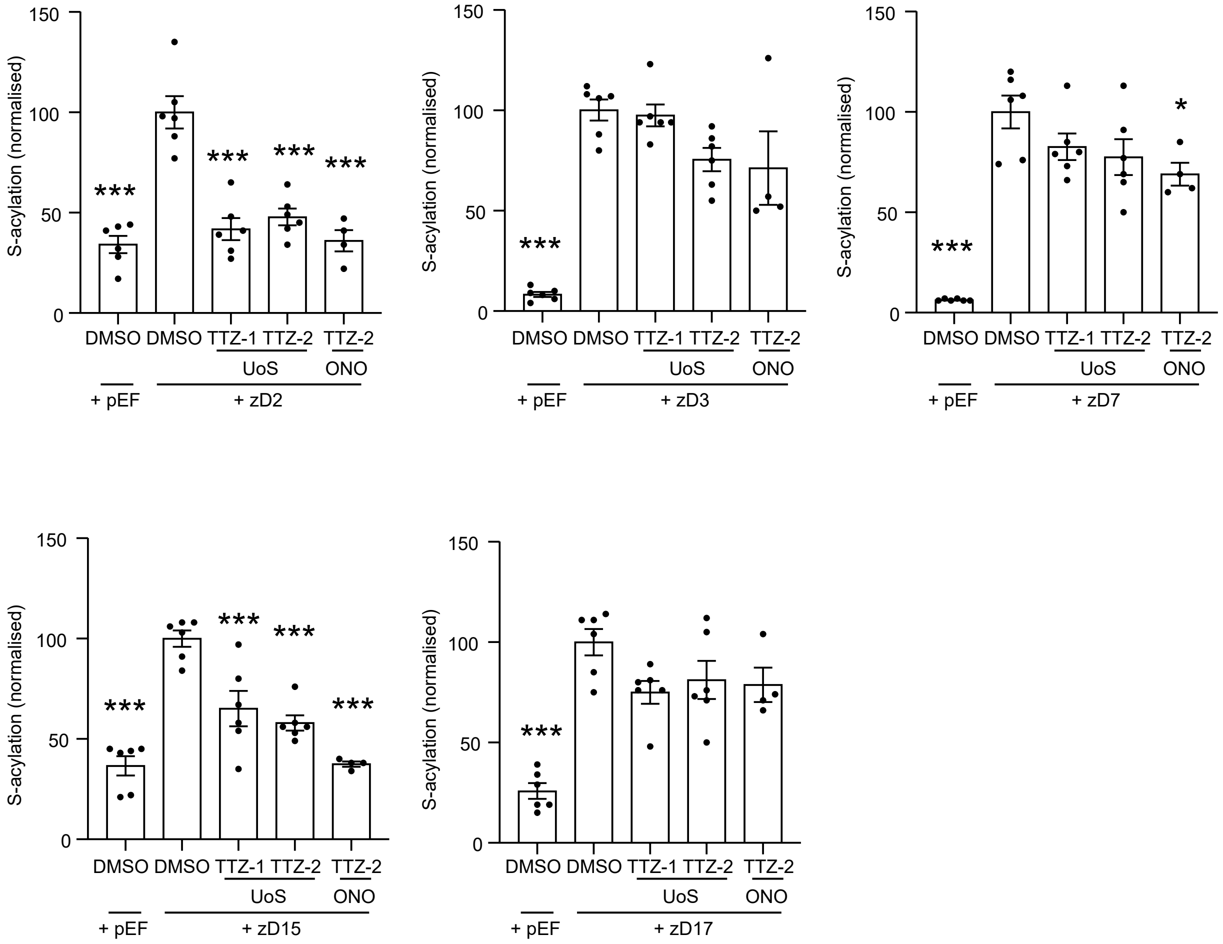

Supplementary Figure 2

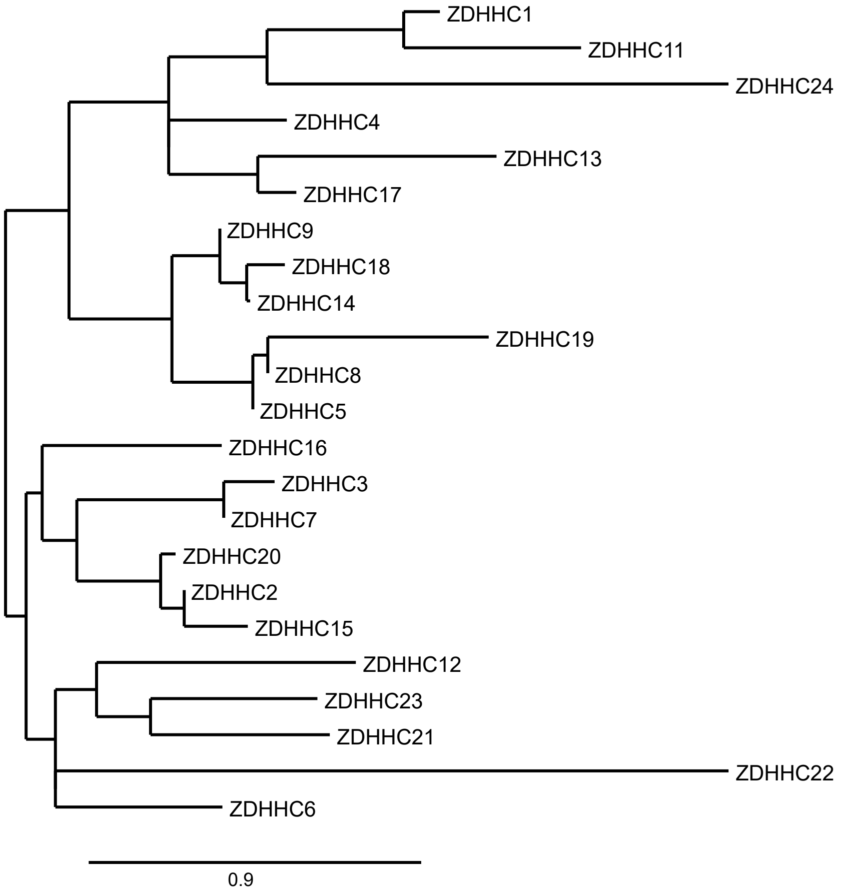

Supplementary Figure 3

***2,6-Dinitroaniline* 6, ^{1}H NMR 500 MHz DMSO *d*$_6$**

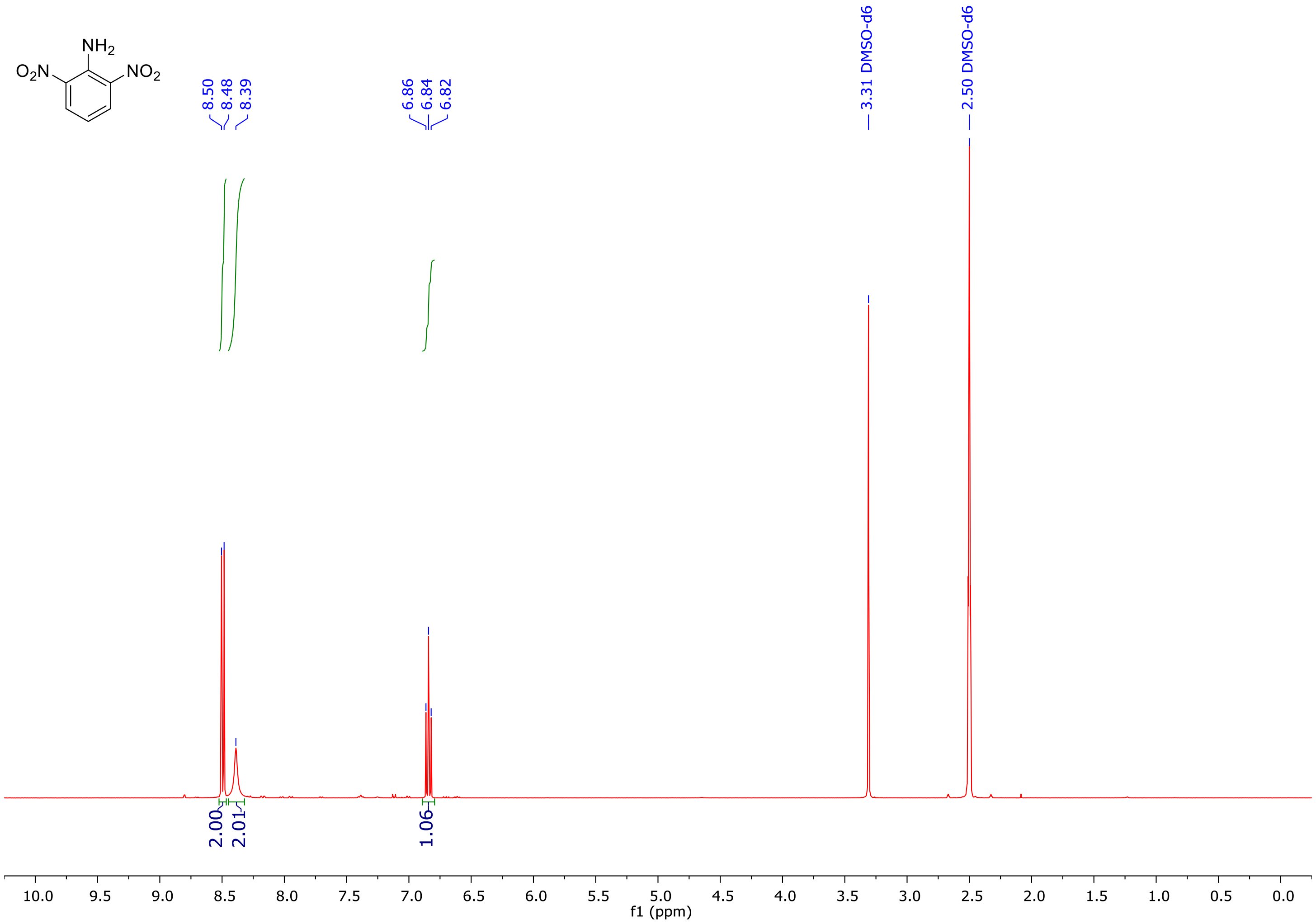

2,6-Dinitroaniline* 6, ^{13}C NMR 126 MHz DMSO *d_6

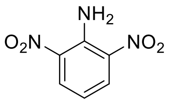

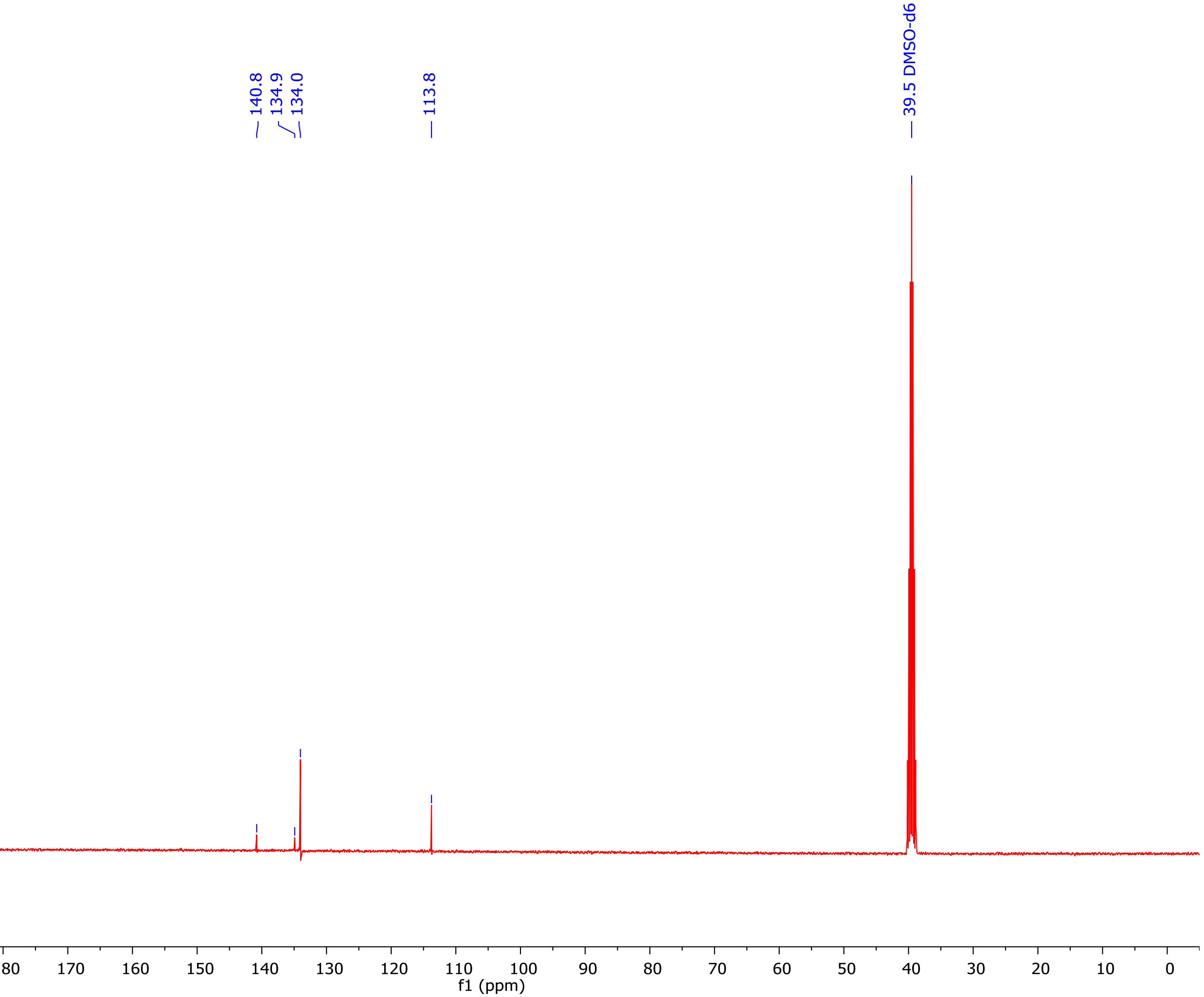

***2-Chloro-1,3-dinitrobenzene* 7, ¹H NMR 500 MHz Acetone *d*₆**

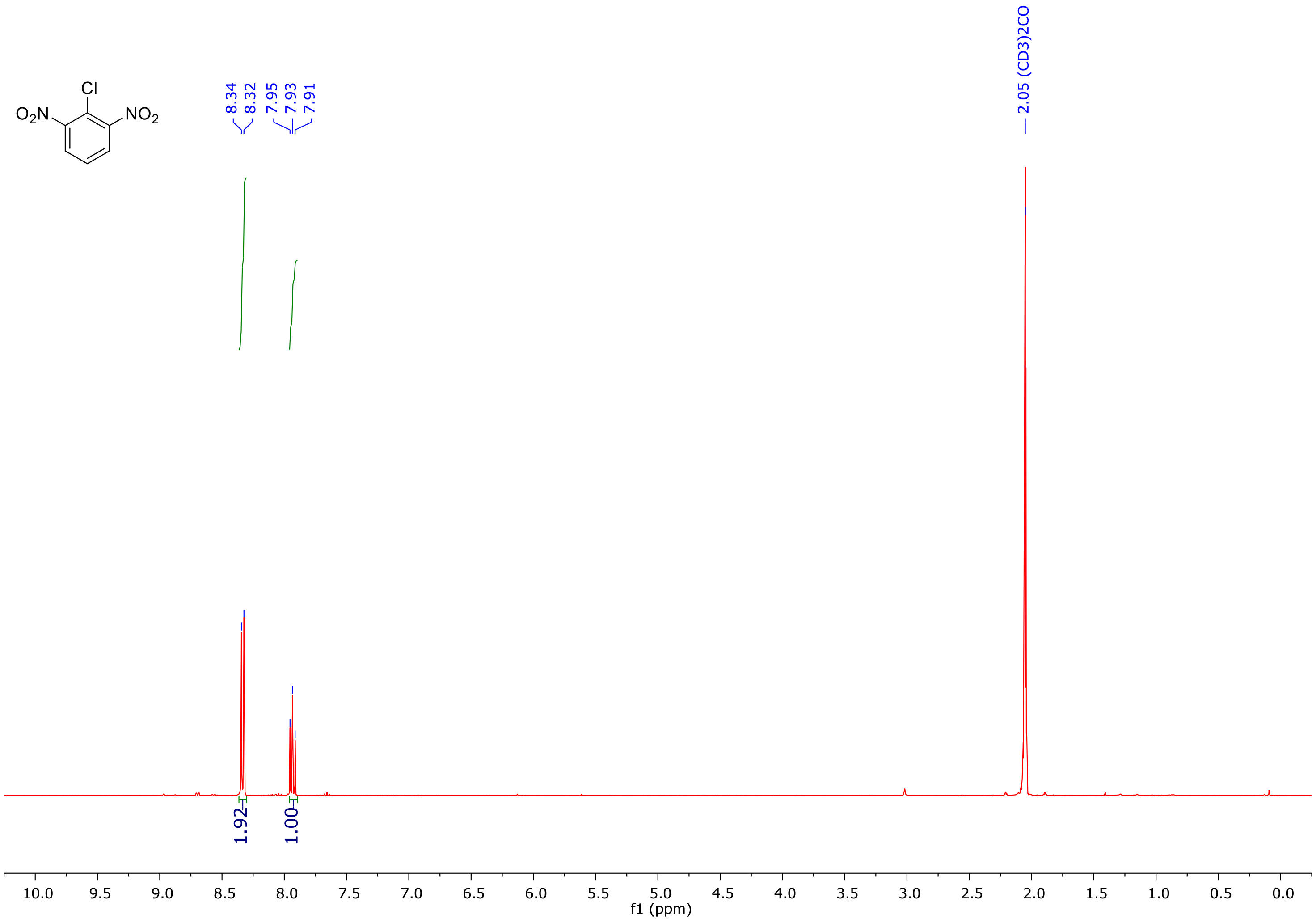

***2-Chloro-1,3-dinitrobenzene* 7, ^{13}C NMR 126 MHz Acetone d_6**

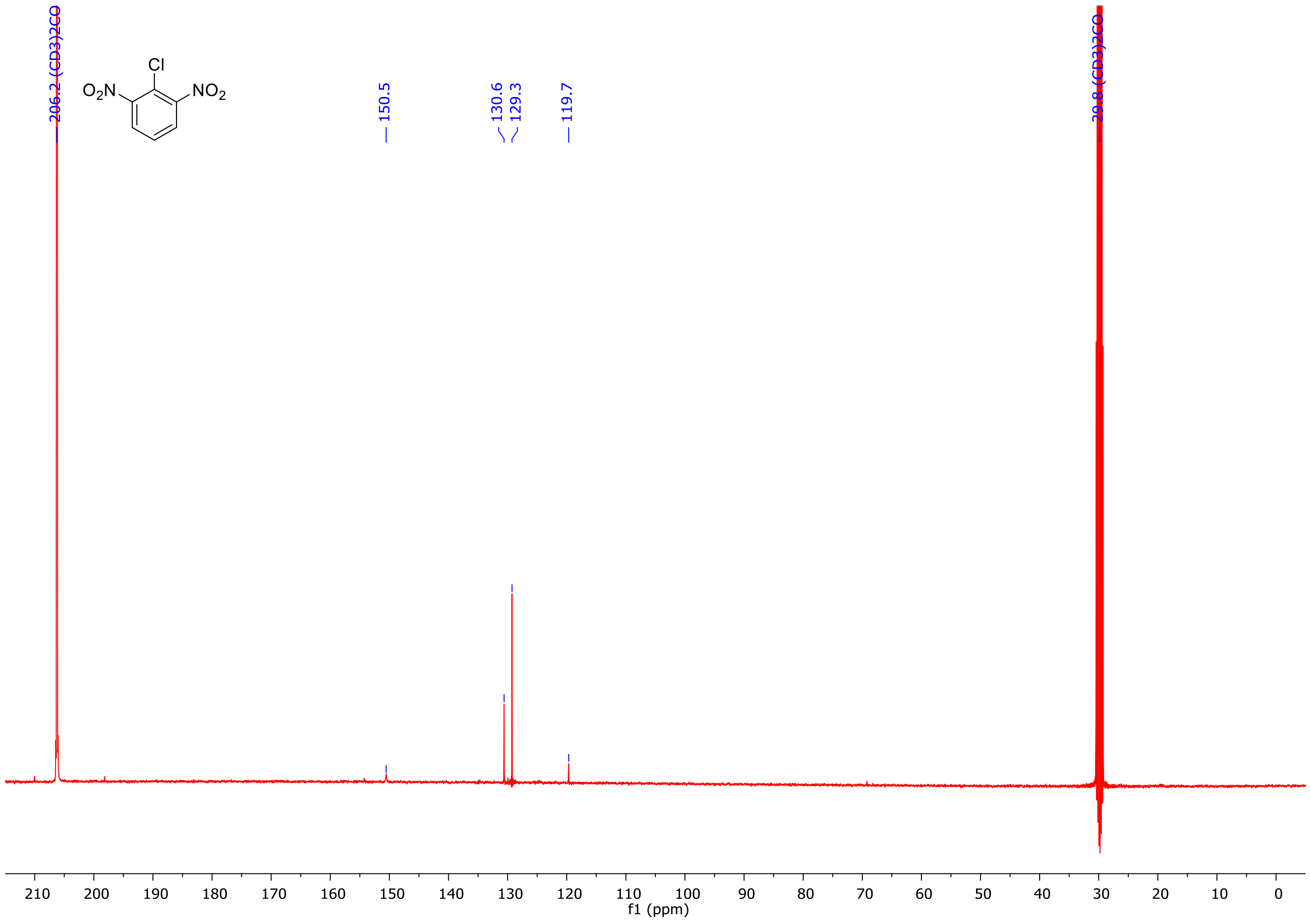

2,6-Dinitrophenol **8, ^{1}H NMR 500 MHz Acetone d_6**

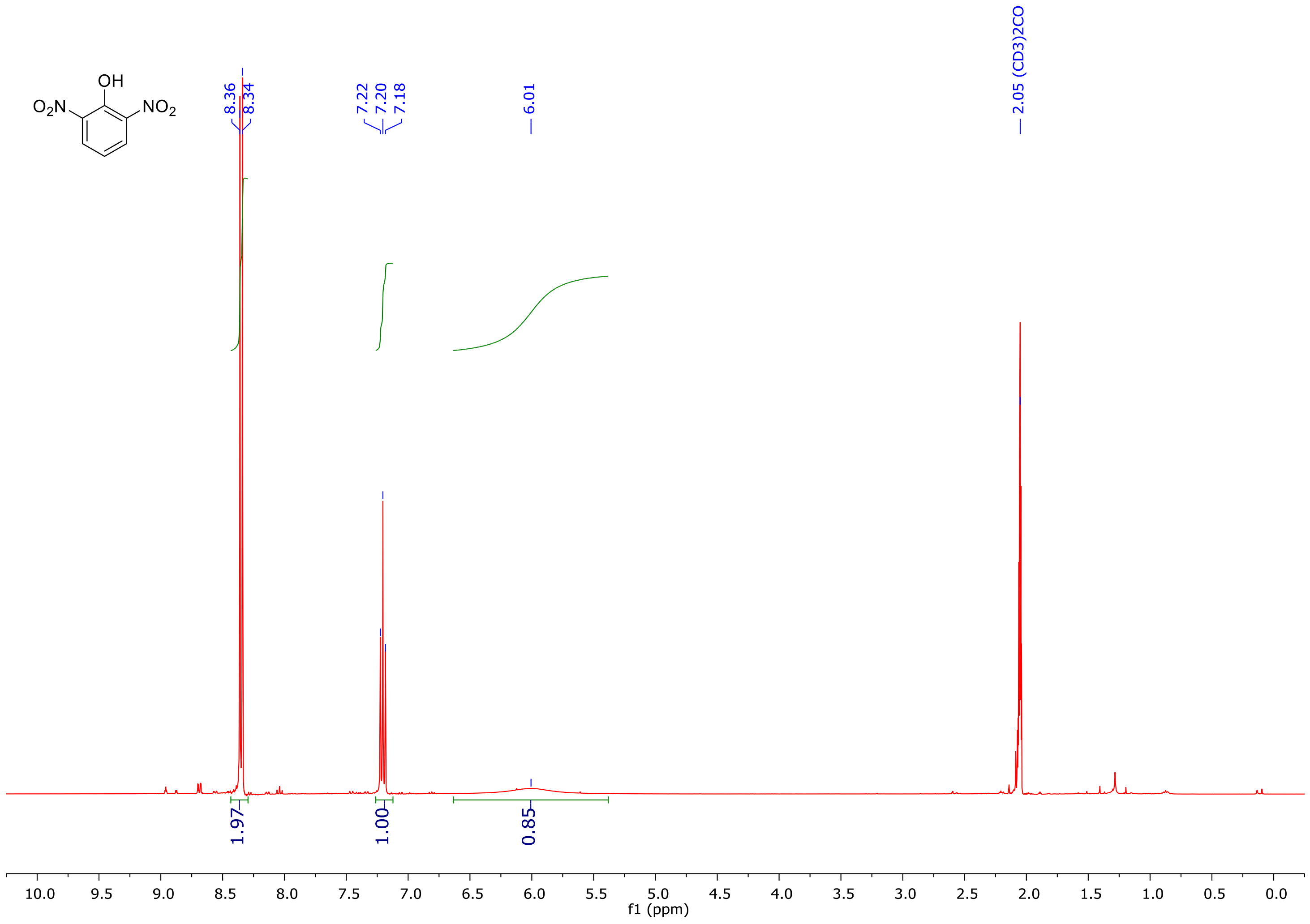

***2,6-Dinitrophenol* 8, ^{13}C NMR 126 MHz Acetone d_6**

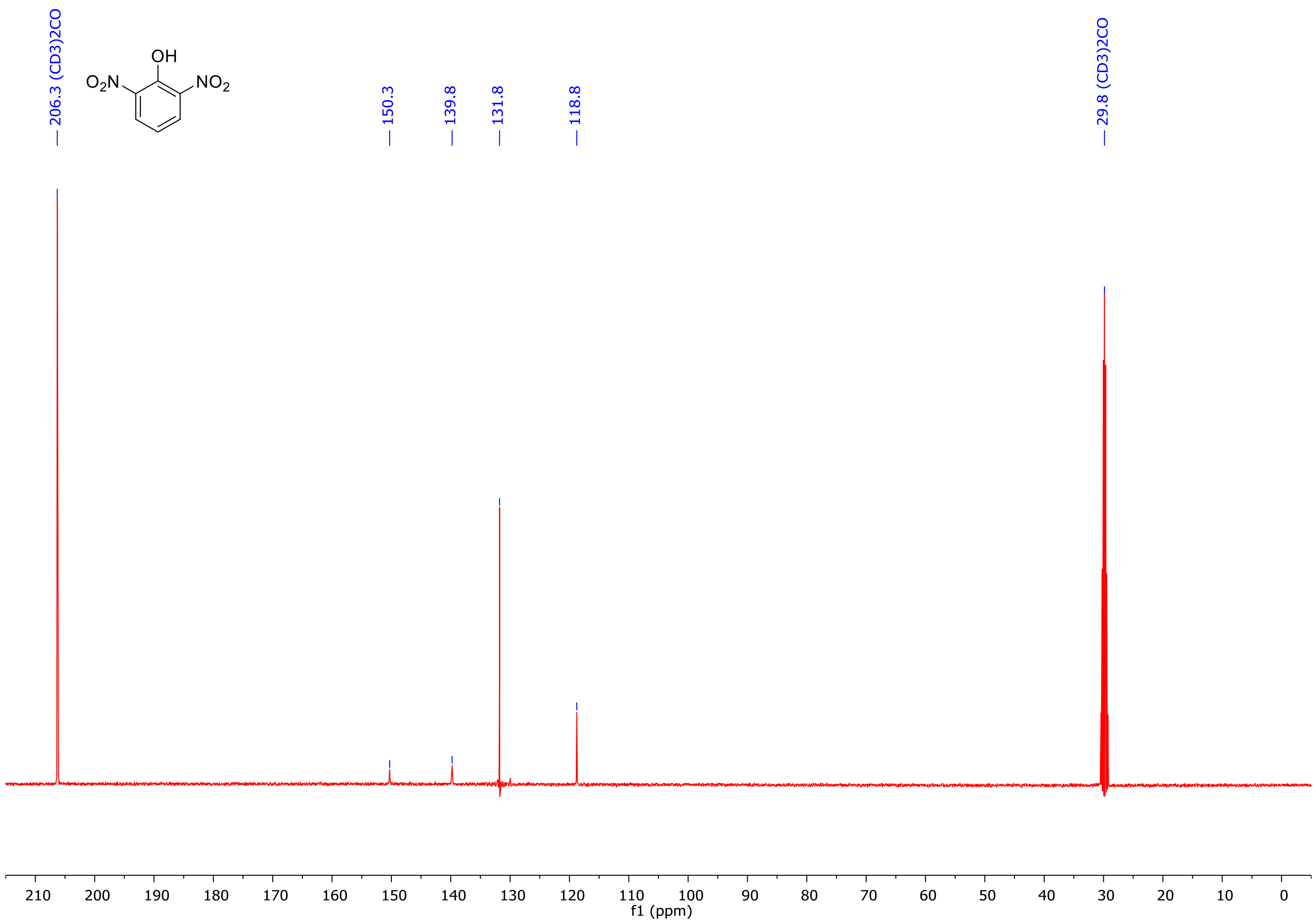

2-Amino-6-nitrophenol* 9, ^{1}H NMR 500 MHz Chloroform *d

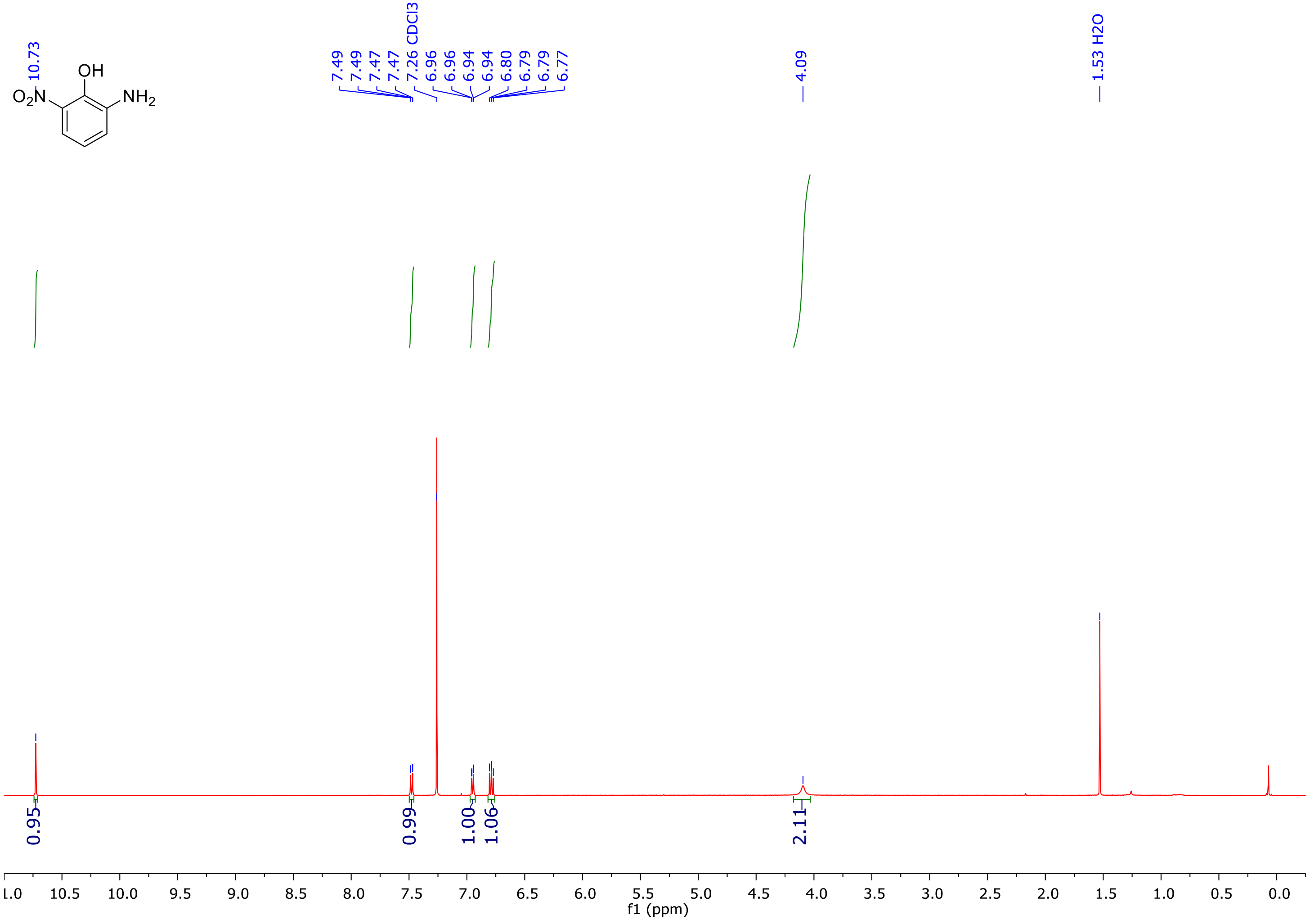

2-Amino-6-nitrophenol 9, ^{13}C NMR 126 MHz Chloroform _d_

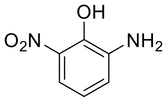

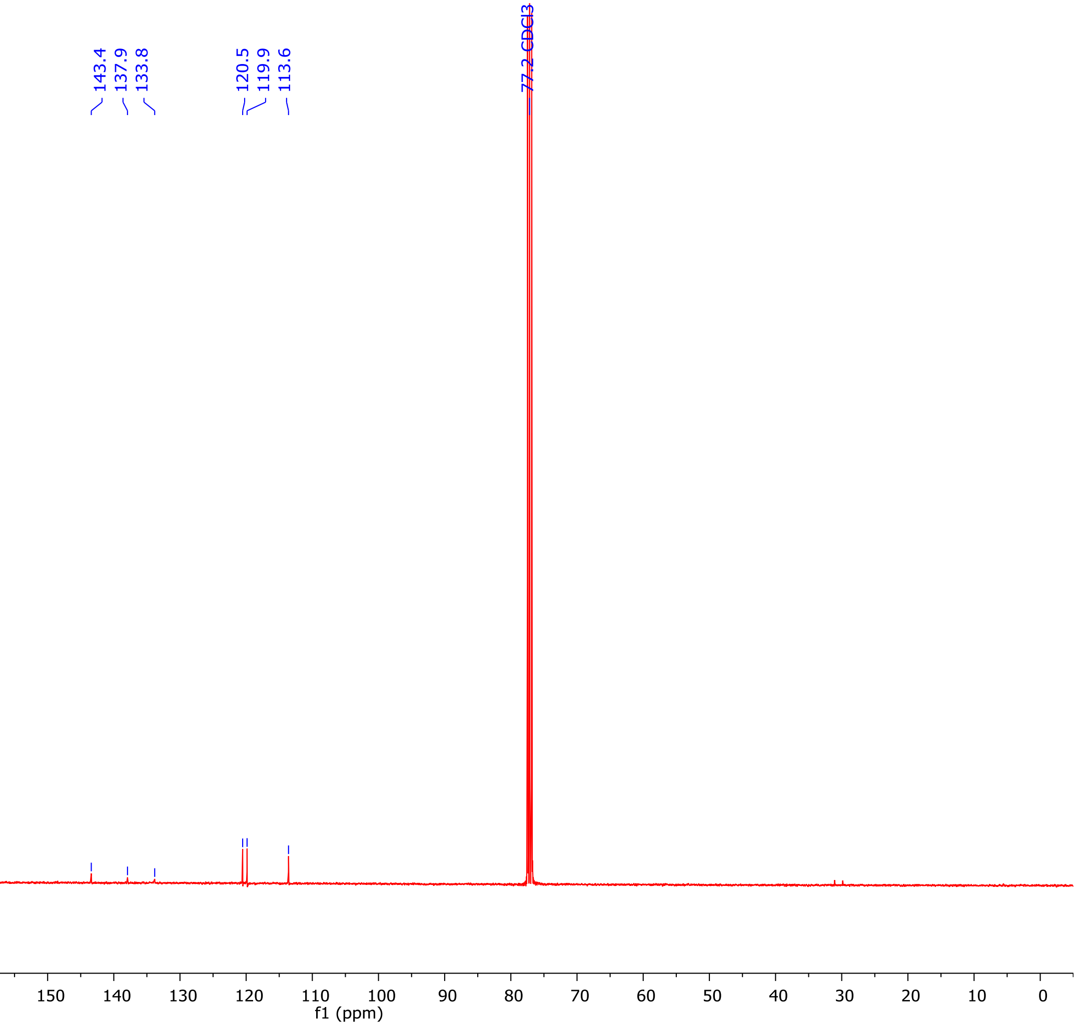

***8-Nitro-3,4-dihydro-2H-benzo[b][1,4]oxazine-2-carbonitrile* 11, ^{1}H NMR 500 MHz Acetone d_6**

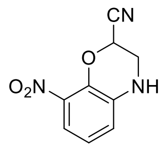

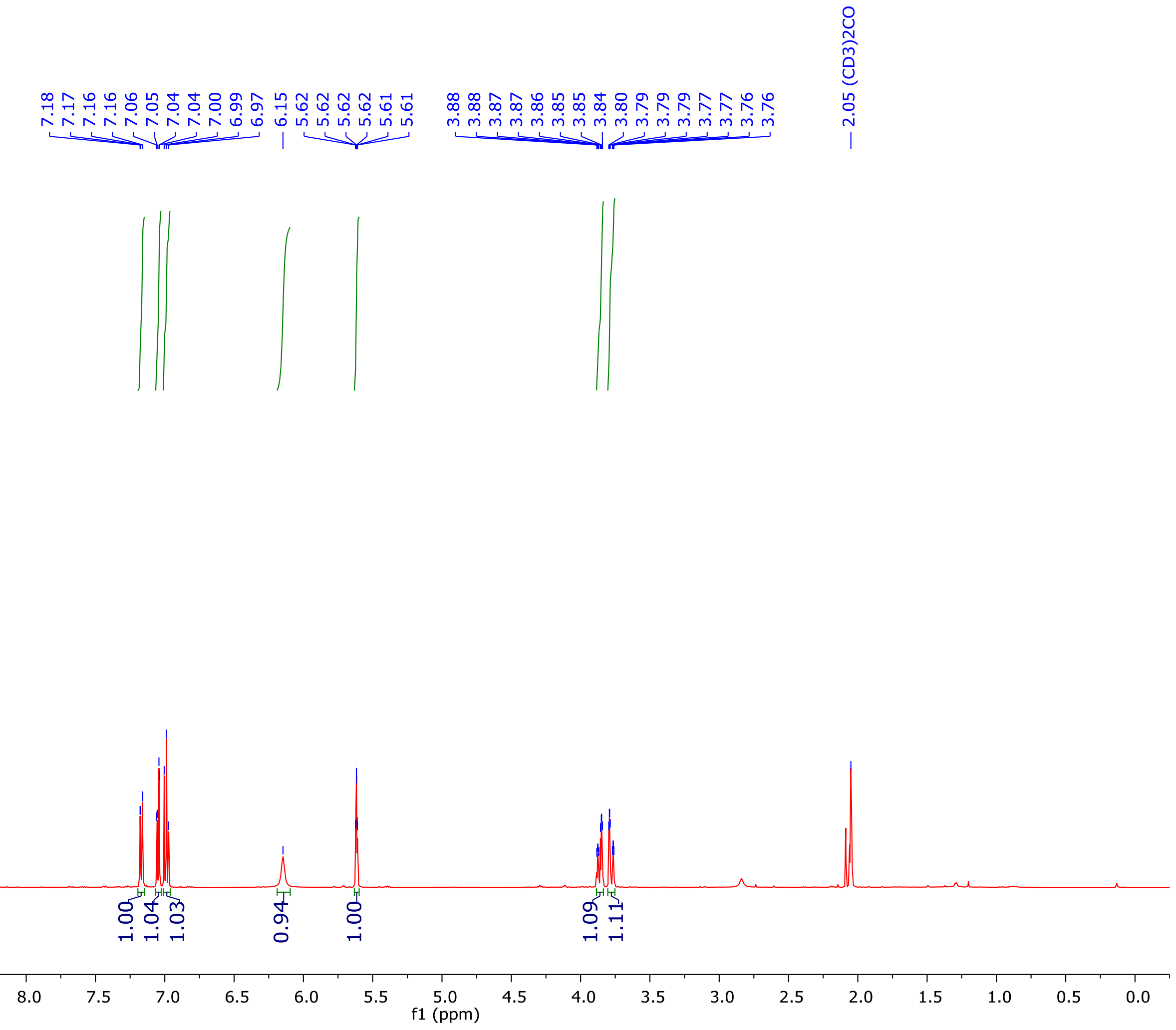

8-Nitro-3,4-dihydro-2H-benzo[b][1,4]oxazine-2-carbonitrile 11, ^{13}C NMR 126 MHz Acetone *d_6*

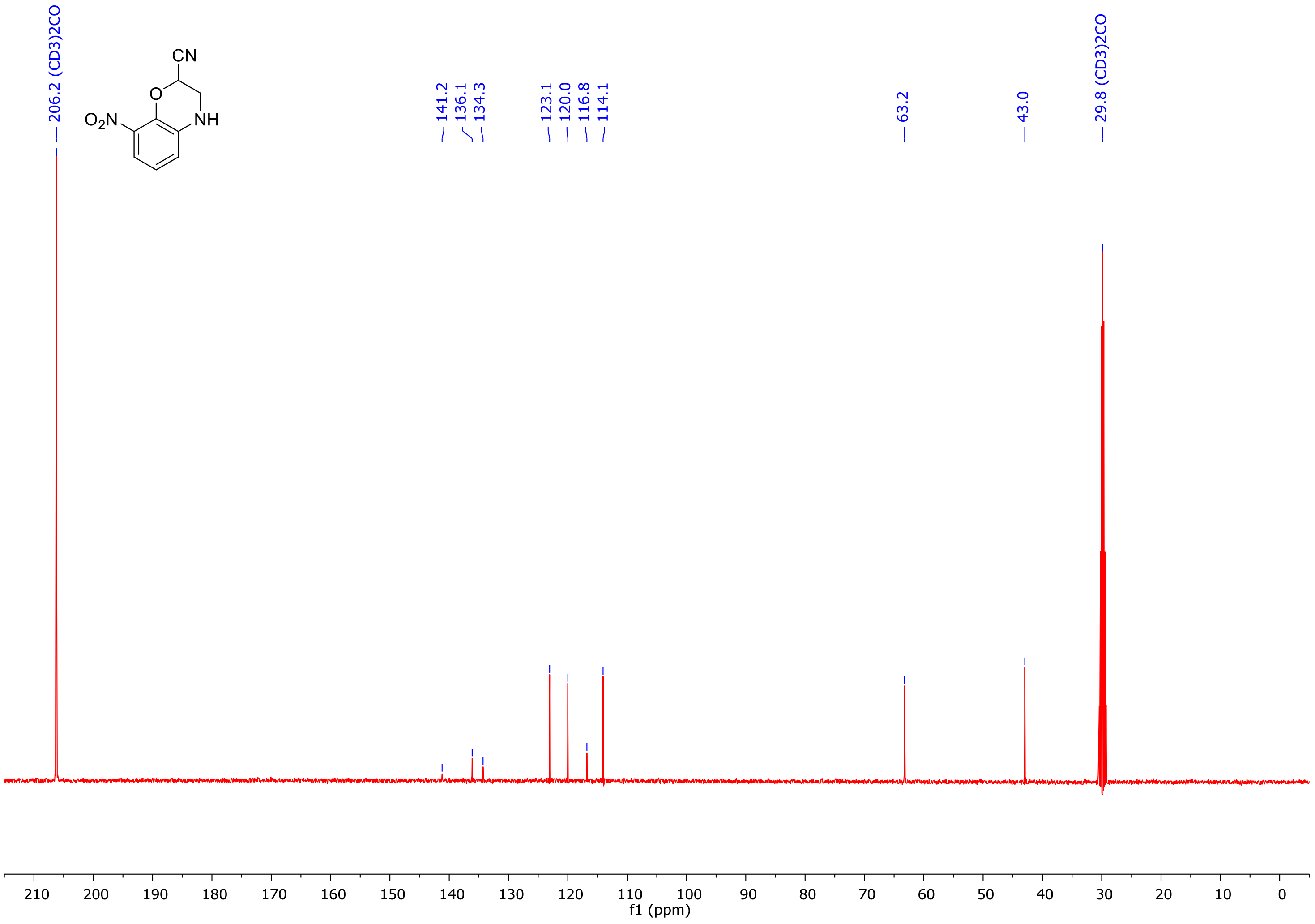

8-Amino-3,4-dihydro-2H-benzo[b][1,4]oxazine-2-carbonitrile **12**, ^{1}H NMR 500 MHz Chloroform *d*

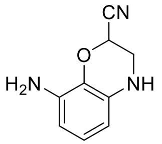

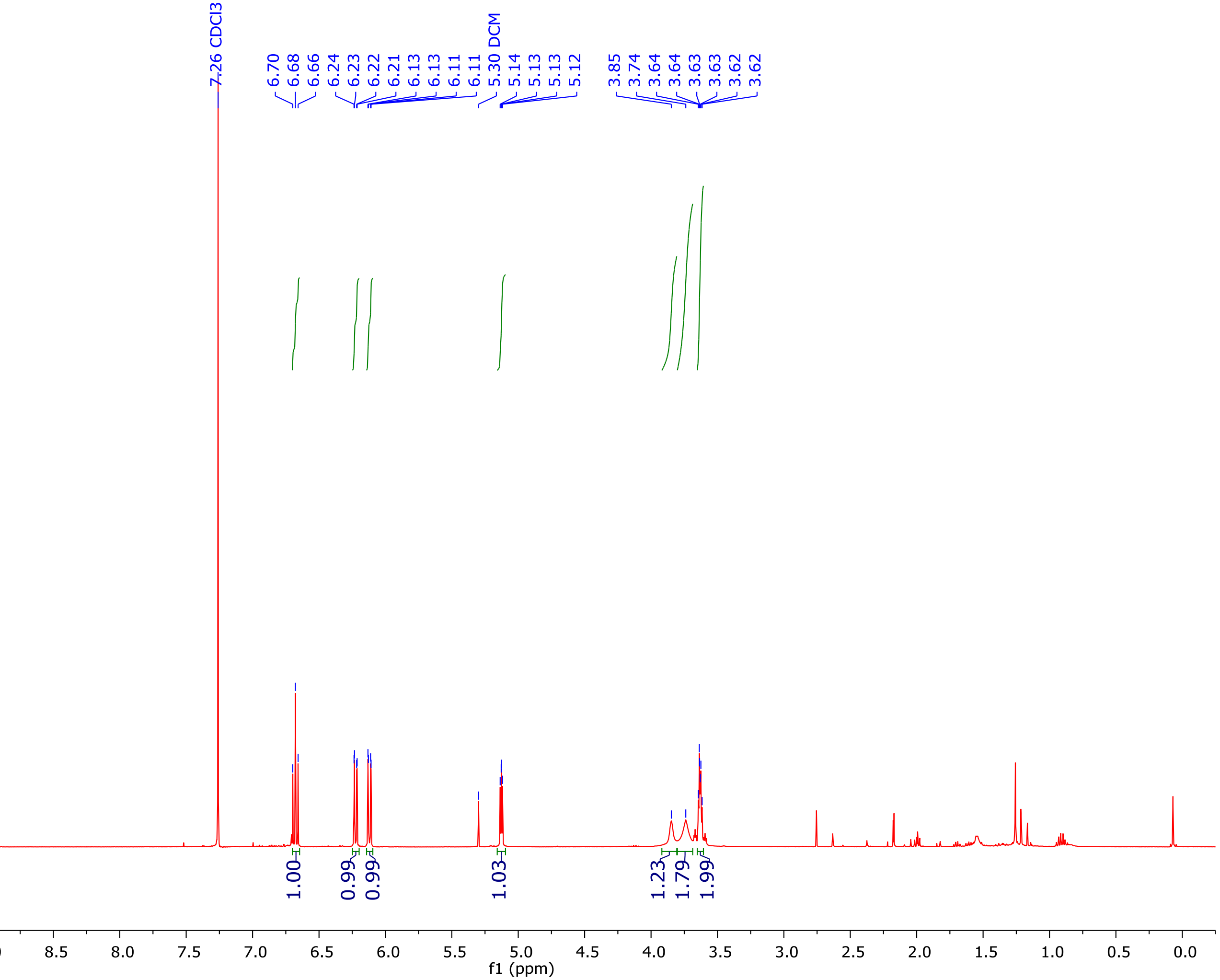

8-Amino-3,4-dihydro-2H-benzo[b][1,4]oxazine-2-carbonitrile 12, ^{13}C NMR 126 MHz Chloroform *d*

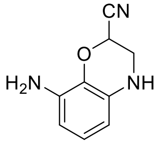

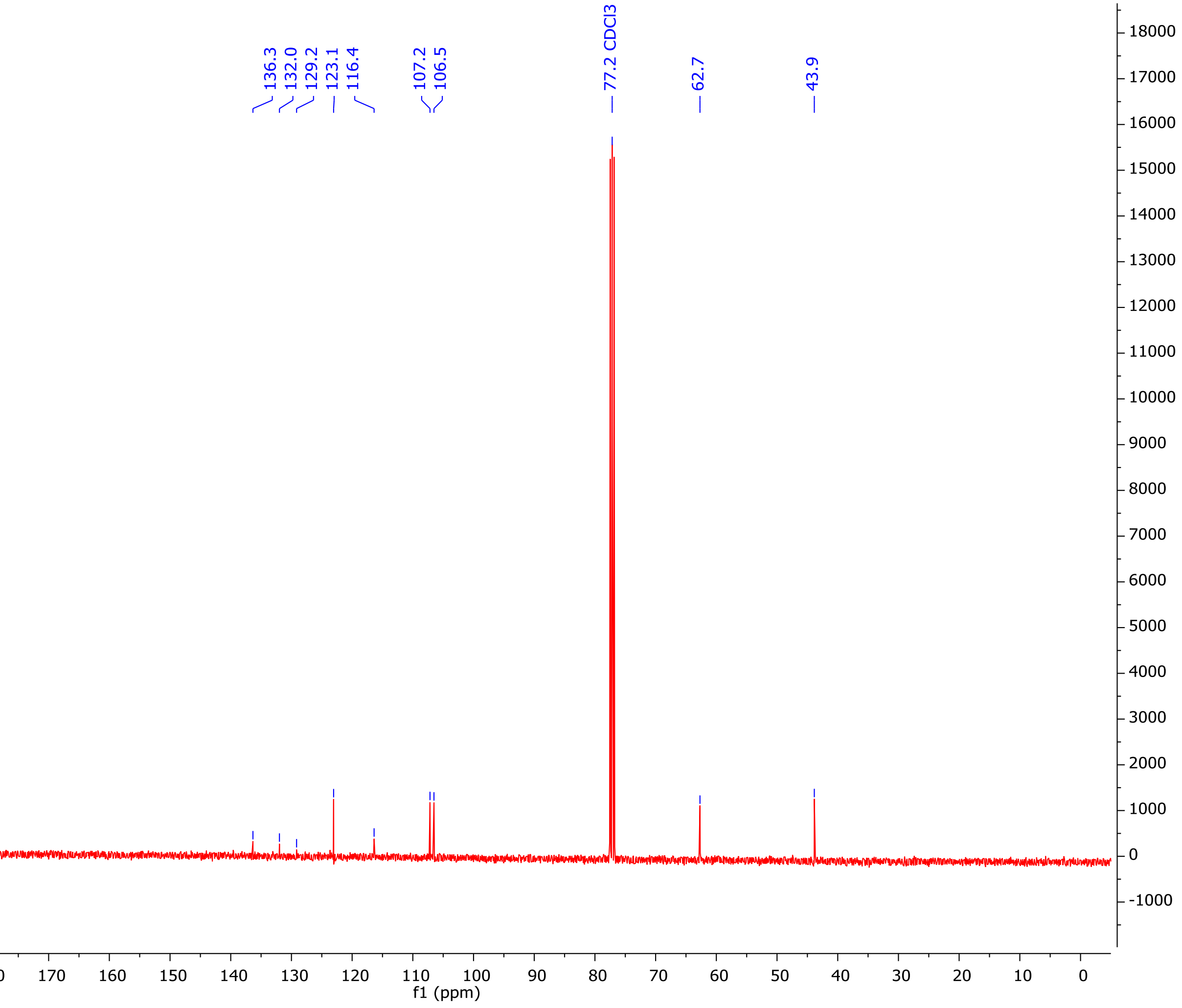

N-(2-Cyano-3,4-dihydro-2H-benzo[b][1,4]oxazin-8-yl)-4-(heptyloxy)benzamide 15, ^{1}H NMR 400 MHz Acetone d_6

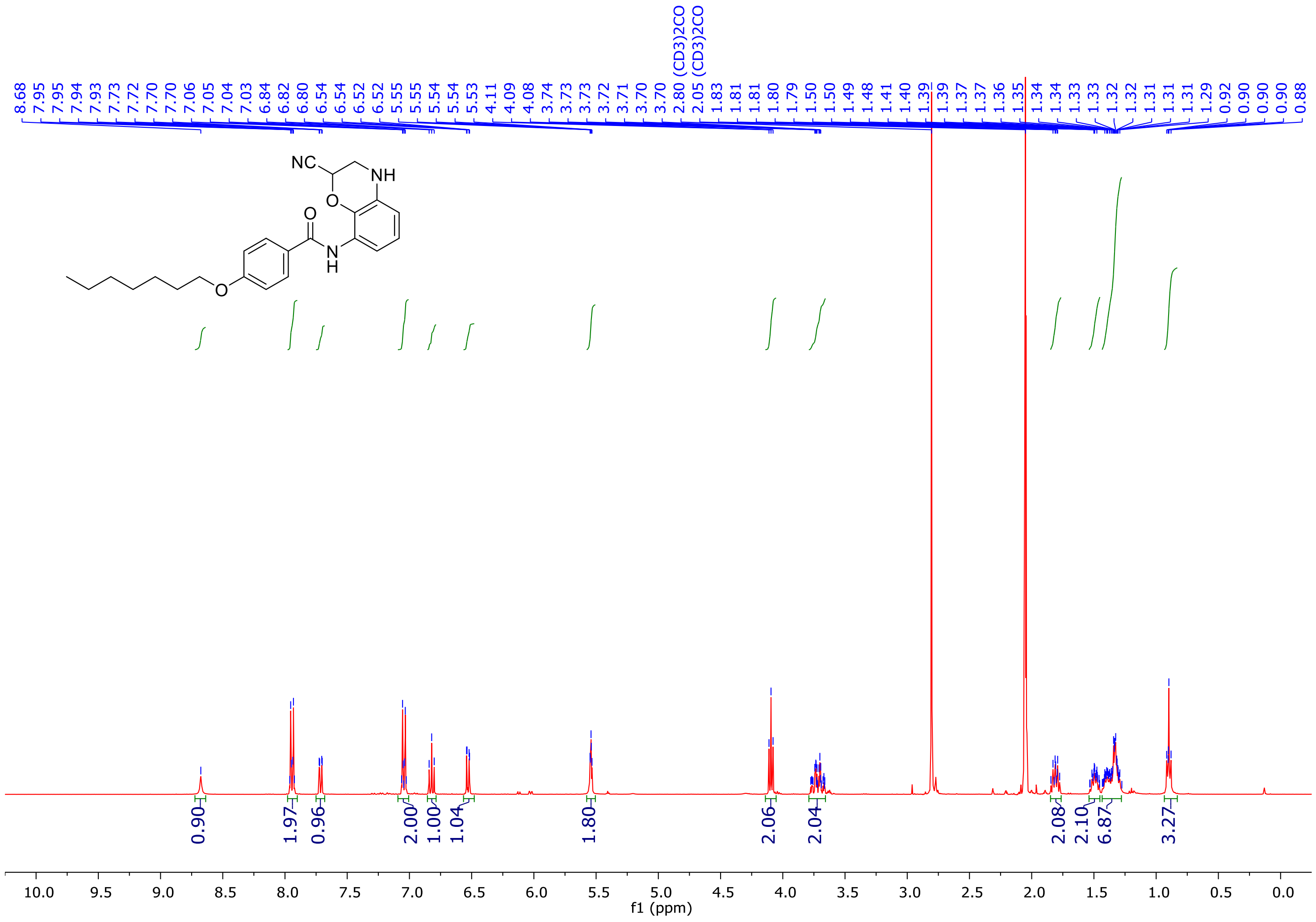

N-(2-Cyano-3,4-dihydro-2H-benzo[b][1,4]oxazin-8-yl)-4-(heptyloxy)benzamide 15, ^{13}C NMR 101 MHz Acetone d_6

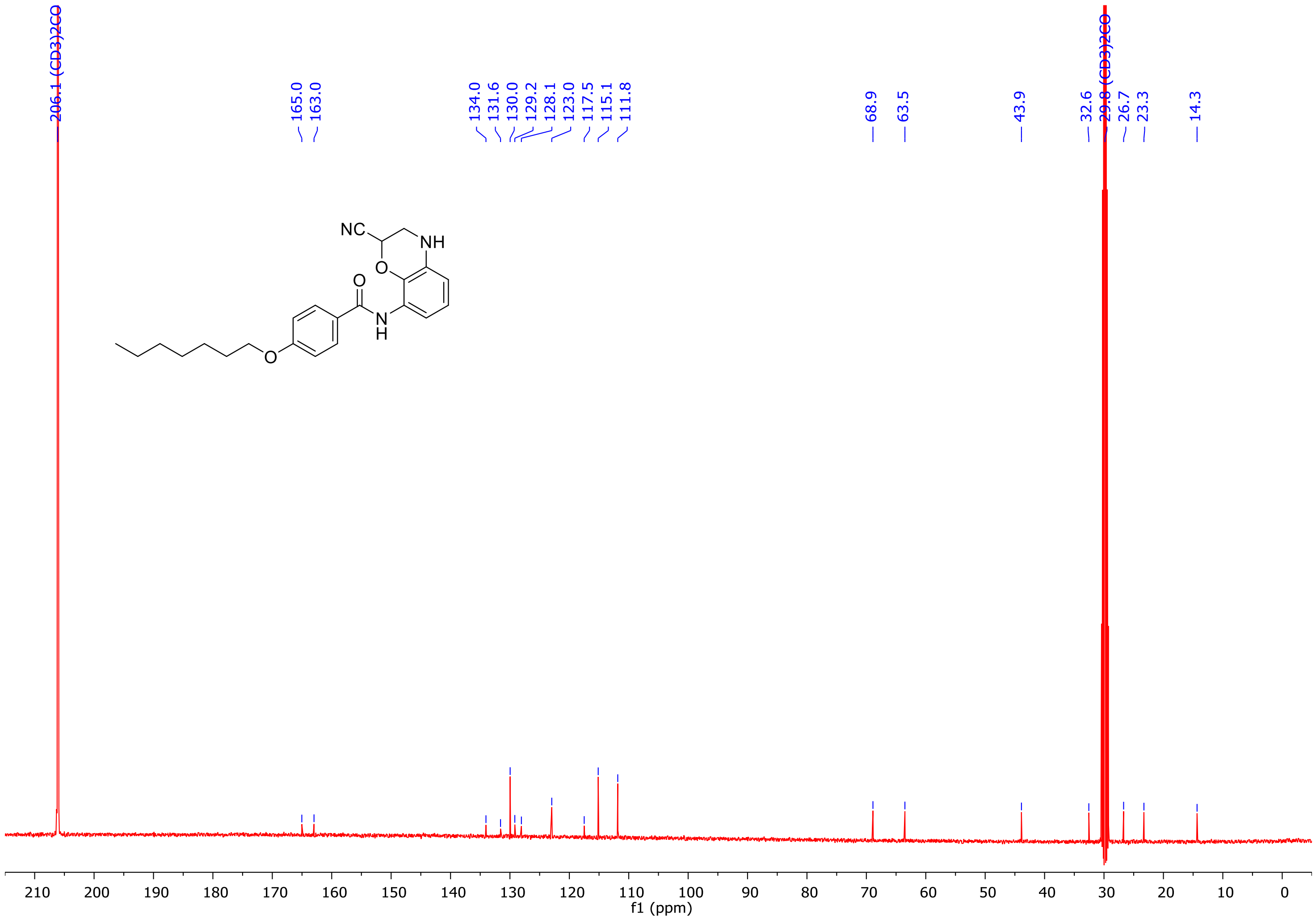

N-(2-Cyano-3,4-dihydro-2H-benzo[b][1,4]oxazin-8-yl)-4-(4-phenylbutoxy)benzamide 16, ^{1}H NMR 400 MHz Acetone d_6

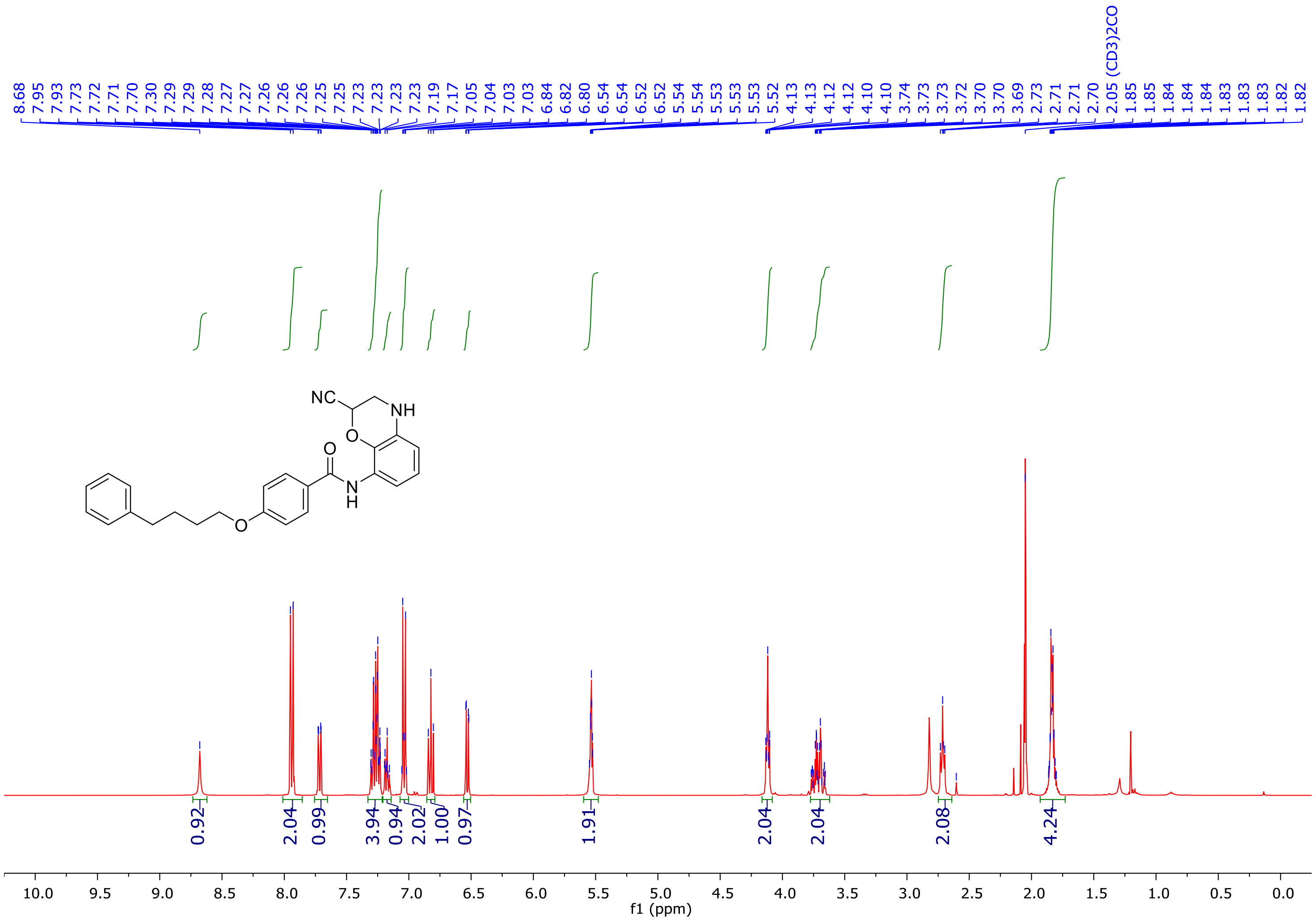

***N*-(2-Cyano-3,4-dihydro-2*H*-benzo[*b*][1,4]oxazin-8-yl)-4-(4-phenylbutoxy)benzamide** **16, ^{13}C NMR 101 MHz Acetone *d*$_6$**

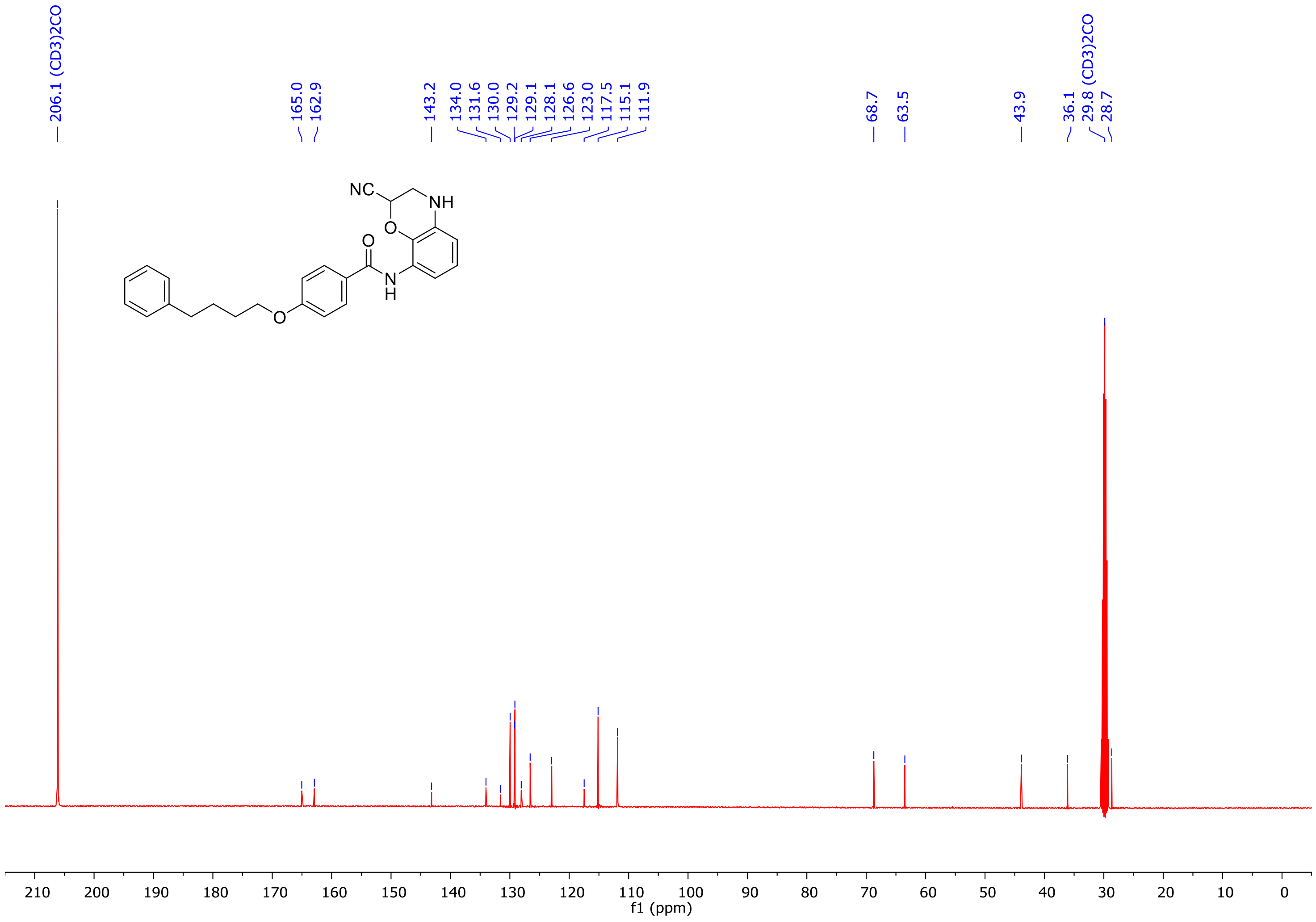

***N*-(2-(1*H*-Tetrazol-5-yl)-3,4-dihydro-2*H*-benzo[b][1,4]oxazin-8-yl)-4-(heptyloxy)benzamide** **TTZ-1 (1), ^{1}H NMR 400 MHz Acetone d_6**

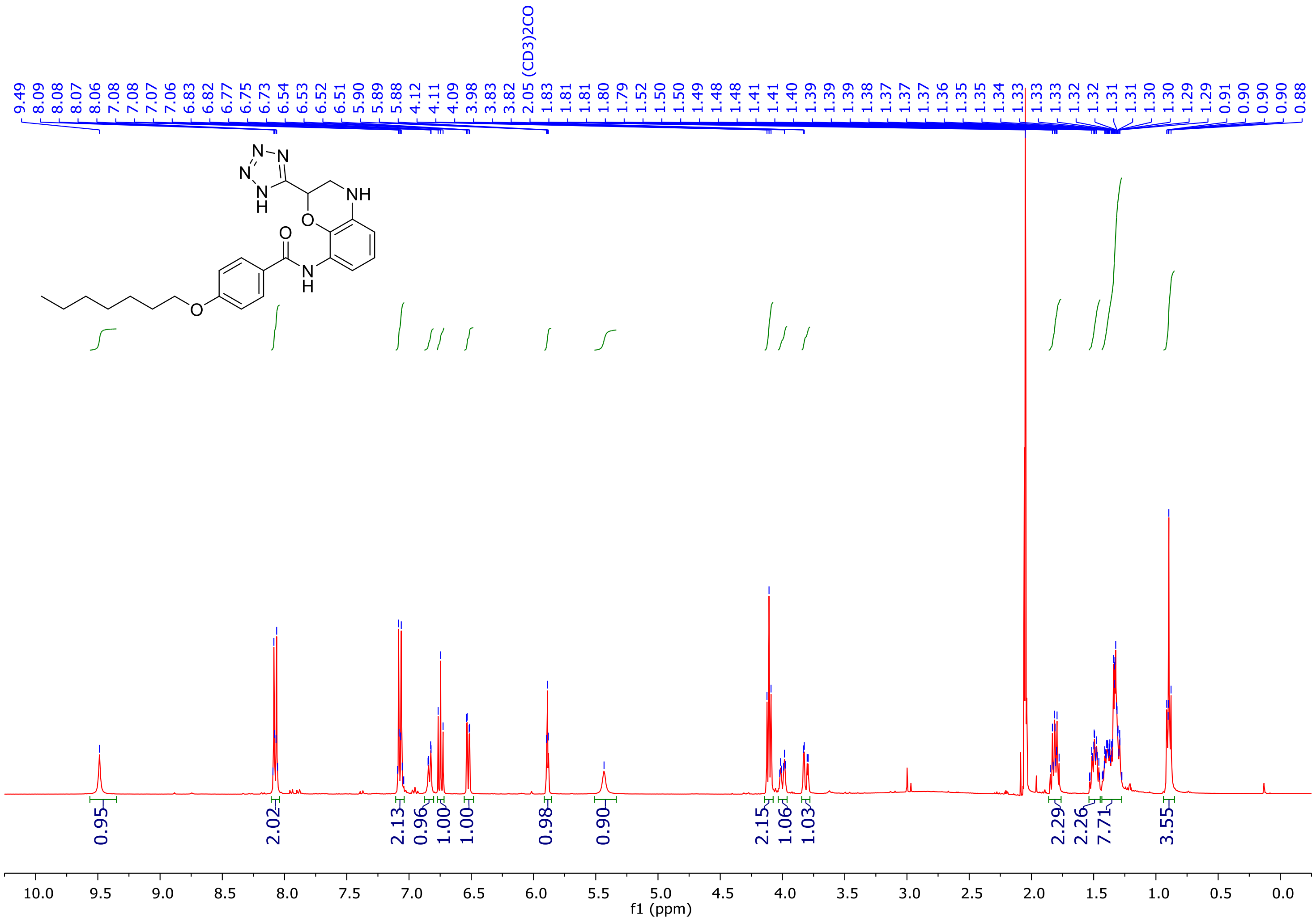

***N*-(2-(1*H*-Tetrazol-5-yl)-3,4-dihydro-2*H*-benzo[b][1,4]oxazin-8-yl)-4-(heptyloxy)benzamide** **TTZ-1 (1), ^{13}C NMR 101 MHz Acetone d_6**

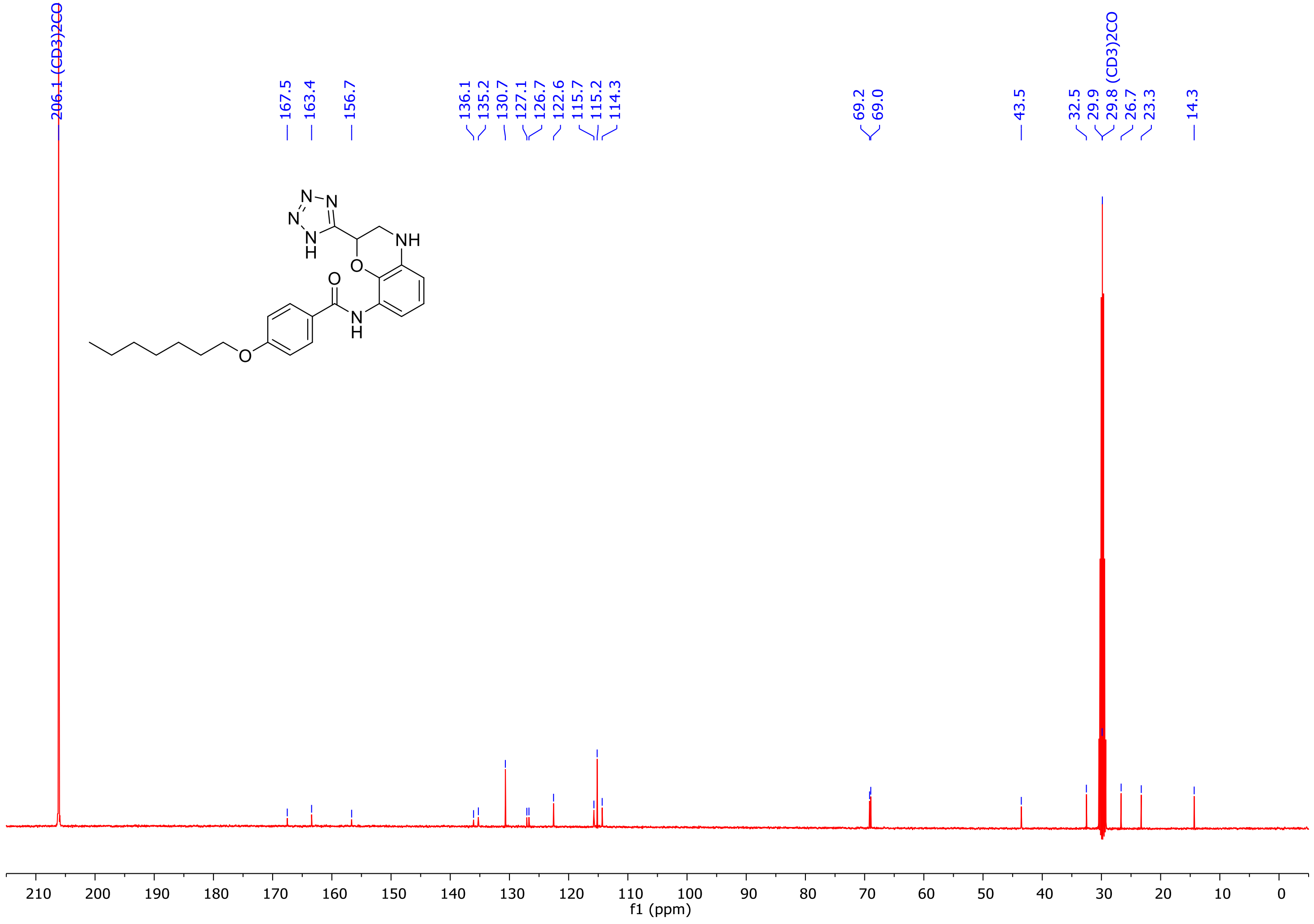

***N*-(2-(1*H*-Tetrazol-5-yl)-3,4-dihydro-2*H*-benzo[b][1,4]oxazin-8-yl)-4-(4-phenylbutoxy)benzamide** **TTZ-2 (2), ^{1}H NMR 400 MHz Acetone d_6**

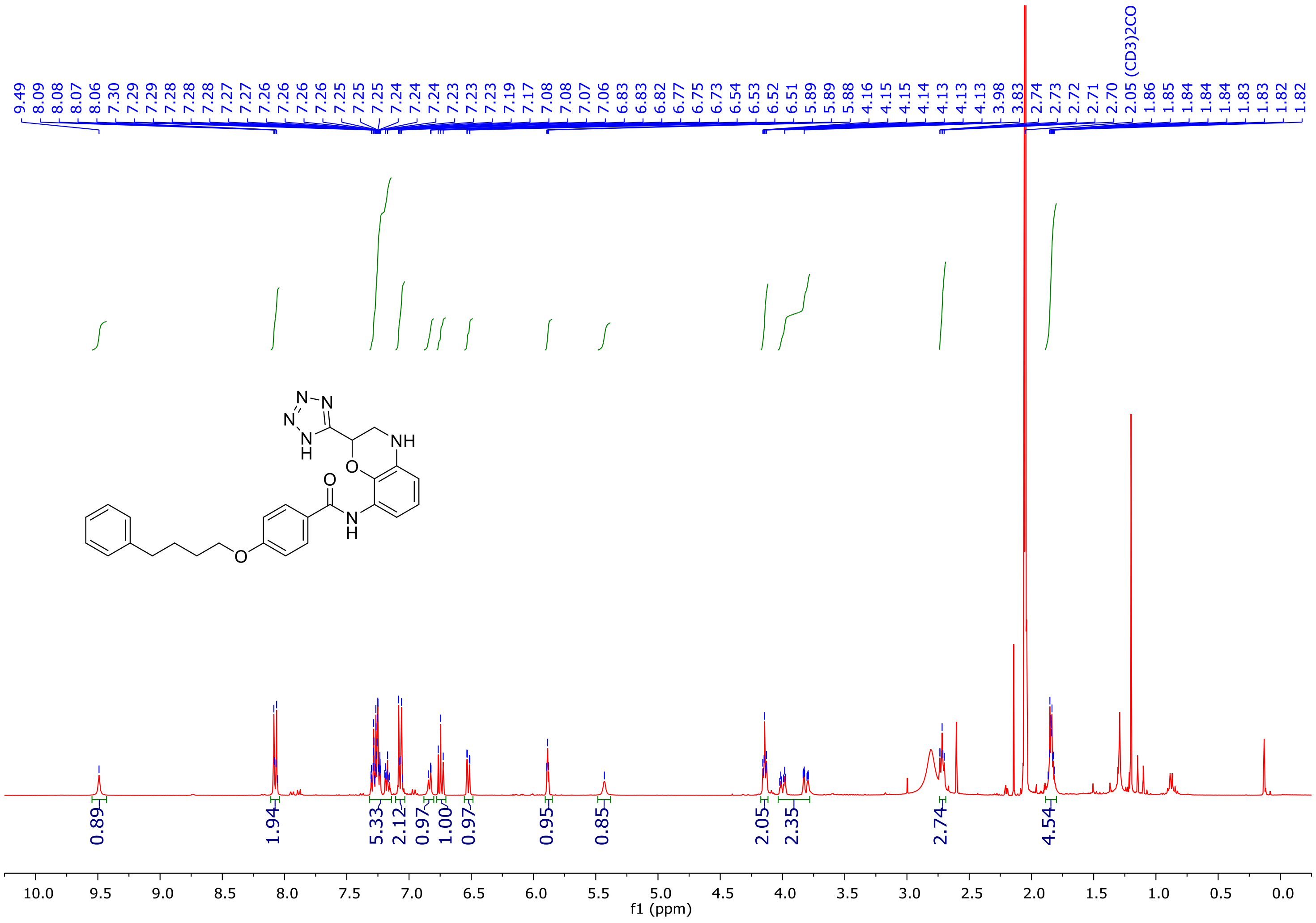

***N*-(2-(1*H*-Tetrazol-5-yl)-3,4-dihydro-2*H*-benzo[b][1,4]oxazin-8-yl)-4-(4-phenylbutoxy)benzamide** **TTZ-2 (2), ^{13}C NMR 101 MHz Acetone d_6**

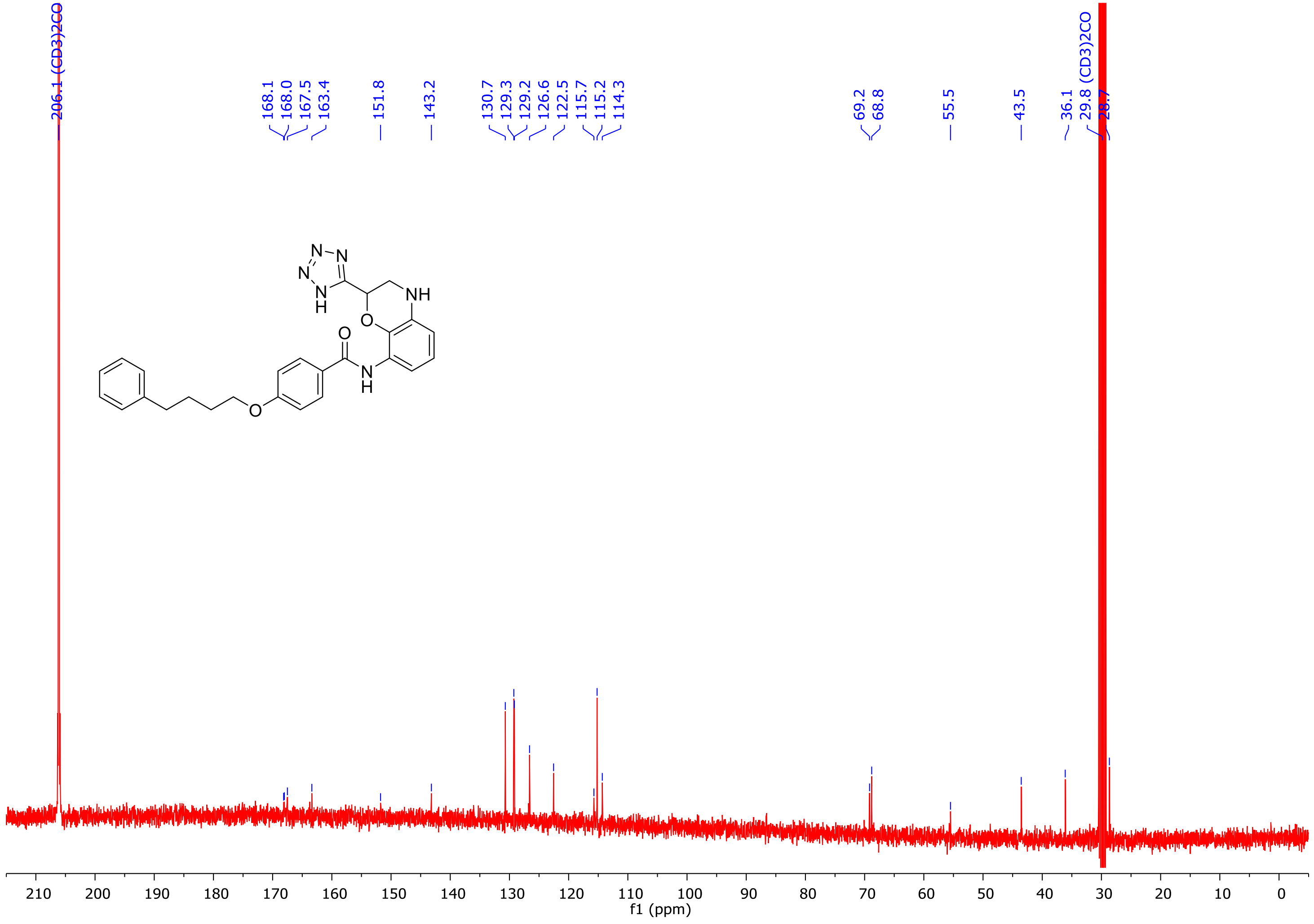

Supplement: Supplemental Figure [file mmc1.pdf]
